# Carotenoids improve the development of cerebral cortical networks in formula-fed infant macaques

**DOI:** 10.1038/s41598-022-19279-1

**Published:** 2022-09-08

**Authors:** Oscar Miranda-Dominguez, Julian S. B. Ramirez, A. J. Mitchell, Anders Perrone, Eric Earl, Sam Carpenter, Eric Feczko, Alice Graham, Sookyoung Jeon, Neal J. Cohen, Laurie Renner, Martha Neuringer, Matthew J. Kuchan, John W. Erdman, Damien Fair

**Affiliations:** 1 Department of Pediatrics, University of Minnesota, Minneapolis, MN 55414 USA; 2 Masonic Institute for the Developing Brain, University of Minnesota, Minneapolis, MN 55414 USA; 3 Center for the Developing Brain, Child Mind Institute, New York, NY 10022 USA; 4 Department of Behavioral Neuroscience, Oregon Health & Science University, Portland, OR 97239 USA; 5 Division of Nutritional Sciences, University of Illinois at Urbana-Champaign, Urbana, IL 61801 USA; 6 Department of Psychology, University of Illinois at Urbana-Champaign, Urbana, IL 61801 USA; 7 Abbott Laboratories, Columbus, OH 43219 USA; 8 Oregon National Primate Research Center, Oregon Health & Science University, Beaverton, OR 97006 USA; 9 Data Science & Sharing Team, National Institute of Mental Health, Bethesda, MD 20892 USA; 10 Department of Food Science & Nutrition and the Korean Institute of Nutrition, Hallym University, Chuncheon, Gangwon-Do Republic of Korea

**Keywords:** Neuroscience, Cognitive neuroscience, Computational neuroscience, Development of the nervous system

## Abstract

Nutrition during the first years of life has a significant impact on brain development. This study characterized differences in brain maturation from birth to 6 months of life in infant macaques fed formulas differing in content of lutein, β-carotene, and other carotenoids using Magnetic Resonance Imaging to measure functional connectivity. We observed differences in functional connectivity based on the interaction of diet, age and brain networks. Post hoc analysis revealed significant diet-specific differences between insular-opercular and somatomotor networks at 2 months of age, dorsal attention and somatomotor at 4 months of age, and within somatomotor and between somatomotor-visual and auditory-dorsal attention networks at 6 months of age. Overall, we found a larger divergence in connectivity from the breastfeeding group in infant macaques fed formula containing no supplemental carotenoids in comparison to those fed formula supplemented with carotenoids. These findings suggest that carotenoid formula supplementation influences functional brain development.

## Introduction

The human brain dramatically increases in size and complexity during the first 2 years of life^1^. One of the developmental processes central to this phenomenon is an extraordinary increase in synaptic density^2–5^. Increased synaptic density combined with selective synaptic pruning^6,7^ underpin the changes in connectivity within and between brain networks. Functional Magnetic Resonance Imaging (fMRI) can be used to measure correlated patterns of brain activity across brain regions. This technique can also be used to characterize functional networks, sets of brain regions with correlated patterns of activity, that are dedicated to tasks such as motor, visual, attention and executive control processes^8^. In this way, fMRI can be used to measure functional maturation of networks in the developing brain.

Breastfed human infants experience more rapid overall brain structural maturation than formula fed infants^9–11^ which can be detected by assessing myelin microstructure^12^. Advantages in both brain structural maturation and cognitive performance positively relate to the extent of breastfeeding^9,11^. Thus, there is a structural basis for the improved cognitive status broadly reported in breastfed infants^13–17^.

The composition of breastmilk differs in a multitude of ways from that of infant formula and may positively impact brain development. For example, lutein and β-carotene are dietary carotenoids routinely found in human milk^18–20^ and lutein is also found in human and macaque breast milk^18–20^, but their concentration in human’s breast milk varies depending on maternal diet^18,19^. Because mammals cannot synthesize carotenoids, formula-fed human and macaque infants have generally lower plasma and tissue lutein levels than breast-fed infants^20–22^. Lutein is selectively concentrated in the macula^23^, a retinal structure uniquely found in humans and higher primates and, importantly, it is the predominant carotenoid in the brain of both human^24^ and macaque infants^20^ despite not being the most common carotenoid in either the diet or breast milk. In contrast, β-carotene is generally found at higher levels in diet but is found less consistently in brain tissue of human and macaque infants and at dramatically lower levels compared to lutein^20,24^. Like lutein, β-carotene is a strong antioxidant, but unlike lutein, it can be converted to vitamin A^25^. Although long term supplementation of adult humans with β-carotene provided benefits in verbal memory and cognition global score^26^, its role in brain development has not been reported.

Lutein intake is associated with positive outcomes in brain health. During pregnancy, maternal lutein consumption leads to offspring with higher scores of verbal intelligence and behavioral regulation in mid-childhood^27^, suggesting an impact on the developing fetal brain. In addition, higher breast milk concentrations of lutein and choline are associated with better recognition memory in 5 month old infants^28^. In 8–10 year old children, macular lutein concentrations are positively correlated with cognition^29^. In randomized supplementation studies, dietary lutein improved measures of visuomotor processing speed in younger adults^30,31^. In older adults, lutein remains the predominant carotenoid in the brain^32,33^ and higher blood levels are associated with improved cognitive status^24,33–40^. Together, these findings indicate that lutein may have an important role in supporting brain development and enhanced cognitive functioning.

Unsupplemented infant formulas contain very low levels of lutein and β-carotene and most formulas are not supplemented to match the higher levels found in human milk^20^. A reasonable supplemented concentration in infant formula is approximately 230 nmol/L lutein which is the 4th quartile value found in breastmilk collected from women consuming a healthy level of green leafy vegetables and fruit^41^. Therefore, we supplemented an infant formula with lutein as part of a carotenoid blend that also contain β-carotene, wherein the lutein level matched those driven by a ‘healthy diet’ in human breastmilk and assessed brain development. Given the recent evidence on the potential role of lutein in cognition, it is important to investigate the role of lutein in brain development under controlled conditions.

Nonhuman primates are uniquely suitable to study the impact of lutein on brain development. In contrast to laboratory animals such as rodents and pigs, they accumulate lutein in macular and brain tissue in a manner similar to humans. In addition, they have cerebral cortical systems that have close counterparts in the human brain, such as those supporting visual and somatosensory processing but also higher order processes such as cognition^42–44^. We have previously shown that supplementation with lutein, *β*-carotene, and lycopene lead to different brain bioaccumulation across the brain with the strongest effect for lutein and only in the cerebellum for *β*-carotene^20^. Hence, in the current study, we hypothesize that a supplemental dietary carotenoid blend including lutein and *β*-carotene would improve brain maturation not only in brain networks related to visual processing but in those supporting cognitive processes in humans. To test this hypothesis we examined brain maturation using functional connectivity via connectotyping^45^ in infant macaques fed formulas differing in carotenoid concentrations. We also studied a reference group of infant macaques fed with breast milk and maternal diet. Typical brain development in this study is defined by the maturation pattern exhibited by the reference group.

## Methods

### Animal and diets

Other results from this cohort of infant monkeys have previously been reported^20,46,47^. All procedures were approved by the Institutional Animal Care and Use Committee of Oregon Health & Science University and carried out in accordance with the Society for Neuroscience Policies on the Use of Animals and Humans in Neuroscience Research, as well as the National Institutes of Health Guide for the Care and Use of Laboratory Animals and the ARRIVE (Animal Research: Reporting of In Vivo Experiments) Guidelines. The health of all infant monkeys was continuously monitored by veterinary staff.

On the day after birth, 24 rhesus macaques (*Macaca mulatta*) of the Indian-origin subspecies were randomized to 1 of 3 diet groups: a reference mixed-diet (MD) group that received a combination of breast milk and maternal diet: Fiber-Balanced Monkey Diet 5000 (LabDiet Inc., St. Louis, MO, USA) (n = 8), or one of two infant formulas (Table 1). The control formula contained no supplemental carotenoids but was supplemented with synthetic vitamin E (*all rac*-α tocopherol (αT)) and docosahexaenoic acid (DHA) (UF; n = 8). The UF carotenoid content was (in nmol/L,) 38.6, 2.3, 21.5, and undetected for lutein, zeaxanthin, *β*-carotene, and lycopene, respectively. In contrast, the supplemented formula was supplemented with carotenoids including lutein, zeaxanthin, *β*-carotene, and lycopene (237, 19.0, 74.2, and 338 nmol/L, respectively), natural vitamin E (*RRR-* αT) and DHA (Similac Advance with OptiGRO, Abbott Nutrition, Columbus, OH) (SF; n = 8). There were no differences in the concentration of total αT among groups; αT’s concentration was assessed biweekly in plasma during the study and in different brain areas at the end of the study. Carotenoids concentrations, in contrast, exhibited large differences among groups^46^. A more complete composition of each diet was previously provided^20,46^.

**Table 1:** Concentration of carotenoids, a-tocopherol and docosahexaenoic acid in infant formulas fed to infant rhesus macaques.

Supplement	Supplemented formula (SF)	Unsupplemented formula (UF)
Lutein (nmol/L)	237	38.6
Zeaxanthin (nmol/L)	19.0	2.3
β-carotene (nmol/L)	74.2	21.5
Lycopene (nmol/L)	338	Undetected
α-Tocopherol (μmol/L)	20.9	29.7
α-Tocopherol (IU/L)	12.2	12.8
Docosahexaenoic acid (nmol/L)	18.9	18.9

Formula-fed infants were nursery reared by bottle from 1 day after birth according to an established protocol^20^ and received only their assigned infant formula for the 6-mo study period. In contrast, MD infants were housed with their dam and received a mixture of breast milk and their mother’s diet, Fiber Balanced Monkey Diet 5000 (LabDiet) plus a variety of fresh fruits and vegetables. As required, MD infants were not restricted from eating the maternal diet. The infants generally began ingesting small amounts of monkey diet at approximately 2 months of age then progressively increased their daily intake to approximately 75–90 g of monkey diet and 60 mL breast milk at 6 months of age^46^. Infant formulas were labeled with a numerical code by Abbott Nutrition, to which all study staff were blinded until analyses were complete. Randomization was stratified by gender and birth weight as previously described^20^, ending up with 4 females and 3–4 males per group. There were no differences in days of age at sacrifice, final body weight, rate of weight gain or formula intake among the 3 dietary groups^20^.

One UF monkey died before 2 months of age. Additionally, scans from 2 UF infants did not pass quality checks due to imperfect delineation of gray and white matter or insufficient data for functional connectivity analysis. As the longitudinal analysis required data from each time point, the final sample size was 21 monkeys with 8, 8 and 5 participants in the MD, SF and UF groups, respectively.

Longitudinal fMRI data were collected at 2, 4 and 6 months of age. The dietary intervention period ended when the infants were 6 months of age, at which time the infant macaques were euthanized under pentobarbital anesthesia by a veterinarian as recommended by the Panel on Euthanasia of the American Veterinary Medical Association. Samples from nine brain areas (prefrontal cortex, occipital cortex, superior temporal cortex, striatum, cerebellum, motor cortex, isolated frontal gray matter, frontal white matter, and hippocampus) were dissected to estimate brain concentration of lutein and β-carotene^20^.

### Imaging acquisition and protocol

Animals were sedated with 5 mg/kg of ketamine and intubated, and anesthesia was maintained with 1% isoflurane vaporized in 100% oxygen. Vital signs were continuously monitored, and homeostasis was maintained. Four T1-weighted (TR = 2500 ms, TE = 3.86 ms; 0.5 mm iso-voxels, 128 slices, FOV = 108 × 128 mm) and one T2-weighted (TR = 10640 ms, TE1/TE2 = 11/95 ms; echo train length = 8, slice thickness = 1 mm, in-plane nominal resolution: 0.5 × 0.5 mm, sampling matrix: 256 × 256, and 60 slices) structural images were acquired for registration. We collected thirty minutes of blood-oxygen-level dependent (BOLD) contrast imaging data using a gradient echo-planar imaging (EPI) sequence (TR = 2070 ms, TE = 25 ms, FA = 90o, 1.5 mm iso-voxels, 32 slices with interleaved acquisition, FOV 96 × 96 mm). A field map scan data (TR = 450 ms, TE = 5.19 ms/7.65 ms, FA = 60°, 1.25 × 1.25 × 2 mm voxels, 40 slices, FOV) was also acquired to correct for image distortions in the BOLD signal.

**Figure 1:**
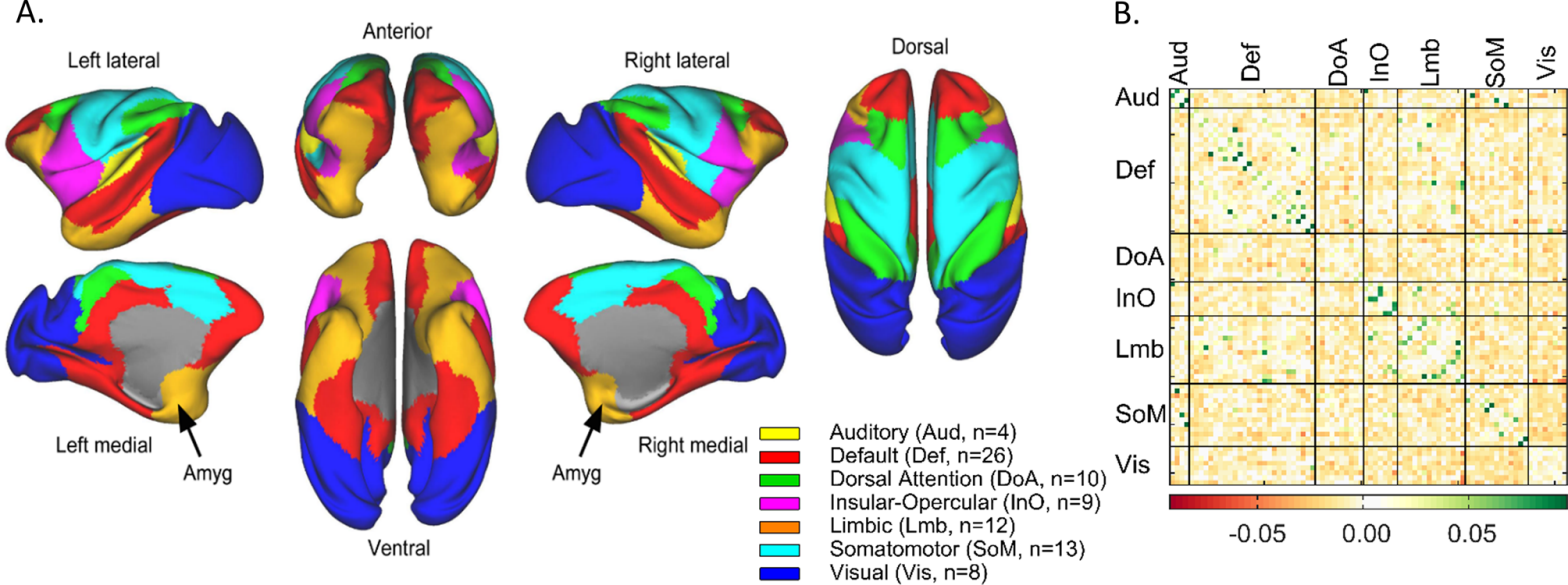
Functional networks and group’s average connectivity matrix. (**A**) Bezgin’s parcellation schema^58^, color-coded by functional network. Color-coded legend also indicates the number of ROIs per network. (**B**) Mean connectivity matrix (n = 63, 3 timepoints from 8 MD, 8 SF and 5 UF macaques) grouped by functional networks.

Data was processed using surface-based registration following the standards and steps proposed by the Human Connectome Project (HCP)^48^, with macaque specific modifications including the use of FSL^49–51^ and FreeSurfer^52–54^. To improve image registration, we incorporated tools from the Advanced Normalization Tools (ANTs)^55^ version 1.9 (http://stnava.github.io/ANTs/) in combination with in-house established tools. Details of this implementation have been described previously by our group^56^. Data were visually inspected to ensure that they were processed and registered properly. We used only BOLD data from low head-movement frames^57^. Resulting BOLD data from each grayordinate of the cortex were parcellated into 82 regions of interest (ROIs) using a predefined macaque cortical parcellation schema^58^ which can be grouped into 7 functional networks^59^ (Fig. 1A). See additional details in Supplementary Materials.

### Functional connectivity

Functional connectivity was calculated using connectotyping^45^ with the parcellated data^58^. Supplementary Fig. 1 shows the mean connectivity values per group and age. The result was a directed connectivity matrix for each scan with 6642 connections (${6642}=82\times \left(82-1\right)$). We symmetrized each matrix ($S=(M+M^{\prime})/2$, where $M$ is a connectivity matrix, $M^{\prime}$ is the transpose of the matrix $M$, and $S$ is the resulting symmetrized matrix) and the resulting matrices had 3321 unique connectivity values. As shown in Fig. 1, connections were assigned to the unique network pair defined by the network each ROI belongs to (Auditory-Auditory, Auditory-Default, …, n = 28)^58^. Count of connections per network pair are reported in the Supplementary Table 1.

### Statistical analysis

We assessed differences in functional connectivity for the factors *group* (MD, SF and UF), *age* (2, 4 and 6 mo), *functional network pair* (n = 28, Auditory-Auditory, Auditory-Default, … See list of all the network pairs in Supplementary Table 1) and their interactions using a 3-way repeated measures ANOVA test in MATLAB. First, a linear mixed effects model was fitted to predict functional connectivity values as a function of *group* using *age* and *functional network* pair as within-subject factors. Next, corrected values were grouped based on between- and within-subject factors, and then a post hoc ANOVA test was used to characterize differences among factors. Mauchly’s test of sphericity was used to test for differences in variance among the groups being compared, and p-values were adjusted accordingly using the correction factor epsilon. Epsilon-adjusted p-values were corrected for multiple comparisons using the Tukey–Kramer method, and 0.05 was used as threshold for significance^60–62^.

As a secondary analysis, we determined if the concentrations of lutein and β-carotene in the brain were associated with functional connectivity using multivariate statistics^60,62–64^. Lycopene did not accumulate in brain despite being included in the diet of supplemented animals. To minimize the risk of overfitting we used regularization via partial least squares regression (PLSR)^65^ to fit models to predict brain carotenoid concentration as a function of functional connectivity, one model for each functional network pair. Significance of the associations was determined by comparing out-of-sample performance using hold-cross association versus null data (N = 4000).

Because carotenoid concentrations^20^ were highly correlated among brain areas we calculated two global indices, one for lutein and another for β-carotene using principal component analysis (PCA). As the first component explained most of the observed variance on each case, we used the first component as a predicted variable. PCA scores were normalized using a logarithmic transformation (Boxcox transformation)^66^, where the logarithmic base was optimized by gradient descent. Associations between lutein and β-carotene indices (PCA scores) and functional connectivity were assessed using imaging data from the last scan (i.e. at 6 mo). We did not attempt to determine causality but rather significant associations. For this reason, it did not matter which variable (carotenoid concentration or functional connectivity) was used as dependent or independent variable.

Model accuracy was estimated by calculating the mean absolute error of the predictions. Performance of the models versus chance was compared by training models using null data for each functional network pair. Models trained with permuted data (4000 times) were also tested with out-of-sample un-permuted data. Significance was determined based on the comparison of the distribution of the mean absolute errors against predicted null-hypothesis data. This comparison was quantified using Cohen’s effect size. Here we report results when predictions had at least a medium effect size (*Cohen’s d* > 0.5). See additional details in Supplementary Materials.

## Results

### Group differences in functional connectivity

Functional connectivity was significantly different among functional network pairs (repeated measures ANOVA test, $F=46.0, p<<0.01$), and for the simultaneous 3-way interaction of the factors: functional network pair, diet, and age (repeated measures ANOVA test, $F=1.5, p=0.002$), as shown in Table 2. No significant main effects were found for diet or age or any other interaction although the interaction between networks and age was close to the threshold for significance (repeated measures ANOVA test, $F=1.3, p=0.08$).

**Table 2:** Repeated measures ANOVA for the effects of group, age, functional network pair and their interactions.

Effect	Sum of squares	Degrees of freedom	Mean squares	F	p-value
Group	0.048	2	0.024	1.0	0.39
Age	0.008	2	0.004	0.3	0.76
Networks	8.629	27	0.320	46.0	**<<1e−6**
Group and age	0.037	4	0.009	0.6	0.64
Networks and group	0.251	54	0.005	0.7	0.97
Networks and age	0.404	54	0.007	1.3	0.08
Networks and group and age	0.916	108	0.008	1.5	**0.002**

Significant values are given in bold.

**Figure 2:**
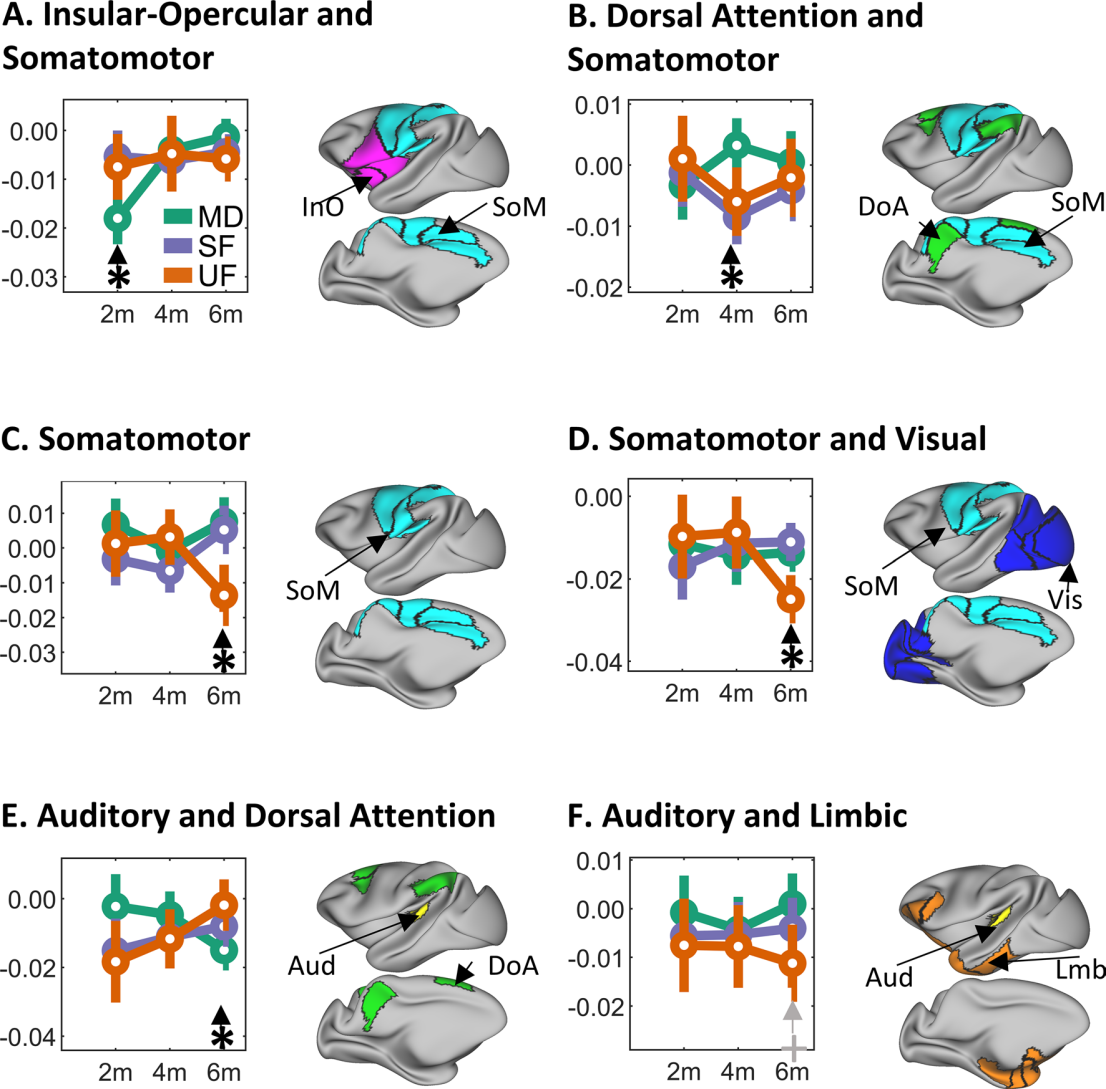
Functional network pairs with significant differences in functional connectivity for the interaction of group (MD, SF and UF) and age (2 m, 4 m, 6 m). Circles represent the mean functional connectivity and the bar indicates the interquartile range. Each plot indicates the *p-value* for each individual repeated measures ANOVA test. Each panel also includes a highly inflated projection of the brain cortex with left-lateral and left-medial views indicating the location of the corresponding functional network pairs.

Following up on the significant 3-way interaction effect, post-hoc analyses revealed significant differences at the 3 ages explored in this study (2, 4, and 6 months), as shown in Fig. 2 and summarized in Table 3. At 2 months of age, group differences were found only for the Insular Opercular—Somatomotor network pair, as shown in Fig. 2A. The MD group had increased negative connectivity compared to the SF group ($difference = 0.013, \;\; p=0.011$, corrected for multiple comparisons), and a trend for increased negative connectivity compared to the UF group ($difference = 0.011, \;\;p=0.067$, corrected), whereas the two formula groups were similar (see Supplementary Table 2 for all the paired comparisons). It is important to note that the large difference between the MD and UF groups is not significant due to the large variability of the data ($difference = 0.016, \;\;p=0.121$, corrected), as reported in Supplementary Table 2.

**Table 3:** Post-hoc analysis for the 3-way interaction of diet, age and networks using functional connectivity.

Functional network pair	Age	Subgroup 1			Subgroup 2			Comparison		
		Diet	Mean fconn	Std. error	Diet	Mean fconn	Std. error	Group 1–group 2	StdErr	p value
InO and SoM	2m	MD	− 0.018	0.003	SF	− 0.005	0.003	− 0.013	0.004	**0.011**
InO and SoM	2m	MD	− 0.018	0.003	UF	− 0.007	0.003	− 0.011	0.004	0.067
DoA and SoM	4m	MD	0.003	0.002	SF	− 0.009	0.002	0.012	0.003	**0.005**
DoA and SoM	4m	MD	0.003	0.002	UF	− 0.006	0.003	0.009	0.004	0.053
SoM and SoM	6m	UF	− 0.014	0.005	MD	0.008	0.004	− 0.021	0.006	**0.005**
SoM and SoM	6m	UF	− 0.014	0.005	SF	0.005	0.004	− 0.019	0.006	**0.011**
SoM and Vis	6m	UF	− 0.025	0.003	SF	− 0.011	0.002	− 0.014	0.004	**0.005**
SoM and Vis	6m	UF	− 0.025	0.003	MD	− 0.014	0.002	− 0.011	0.004	**0.021**
Aud and DoA	6m	MD	− 0.015	0.003	UF	− 0.002	0.004	− 0.013	0.005	**0.038**
Aud and Lmb	6m	UF	− 0.011	0.004	MD	0.001	0.003	− 0.012	0.005	0.072

Each subgroup corresponds to the 3-way interaction of diet, age and networks shown in Fig. 2. Subgroups are sorted by age, then by functional network pair with the lowest p-value. Subgroup 1 has always the largest absolute value of functional connectivity compared to Subgroup 2. p-values < 0.05 highlighted in bold font.

m, age in months; MD, mixed-fed; UF, un-supplemented formula; SF, supplemented formula. InO, Insular-Opercular network; SoM, Somatomotor network; DoA, Dorsal Attention network; Vis, Visual network; Aud, Auditory network; Lmb, Limbic network.

We also found group differences at 4 months of age in the Dorsal Attention and Somatomotor functional network pair (Fig. 2B). The MD group had positive functional connectivity while the formula groups had negative connectivity, with the largest difference between the MD and SF group (*difference* = 0.012, *p* = 0.005, corrected). The difference between the MD and UF was just at the threshold for significance (*difference* = 0.009, *p* = 0.053, corrected). No significant differences were found between the two formula groups. Multiple significant group differences were found at 6 months of age. Within the Somatomotor network (Fig. 2C), the UF was the only group with negative connectivity and was significantly different compared to both the MD group (*difference* = 0.021, *p* = 0.005, corrected) and the SF group (*difference* = 0.019, *p* = 0.011, corrected), whereas values for the SF group were similar to the MD group (Supplementary Table 2 reports all the comparisons). Similarly, in the Somatomotor—Visual network pair (Fig. 2D), the UF group had more negative connectivity compared to both the MD group (*difference* = 0.011, *p* = 0.021) and the SF group (*difference* = 0.014, *p* = 0.021, corrected) at 6 months of age, whereas the SF group did not differ from the MD group. For the Auditory—Dorsal Attention network pair at 6 months (Fig. 2E), the UF group had null connectivity ($mean \; functional \; connectivity = -0.002, \; standard \;error=0.004$) and was significantly different from the MD group that had negative connectivity (*difference* = 0.013, *p* = 0.038, corrected). We found no other differences.

Finally, in the Auditory and Limbic network pair (Fig. 2F), the UF trended to negative connectivity compared to the MD group (*difference* = 0.012, *p* = 0.072, corrected), with no differences among all the other groups.

### Composite index of lutein and  β-carotene concentrations

**Figure 3:**
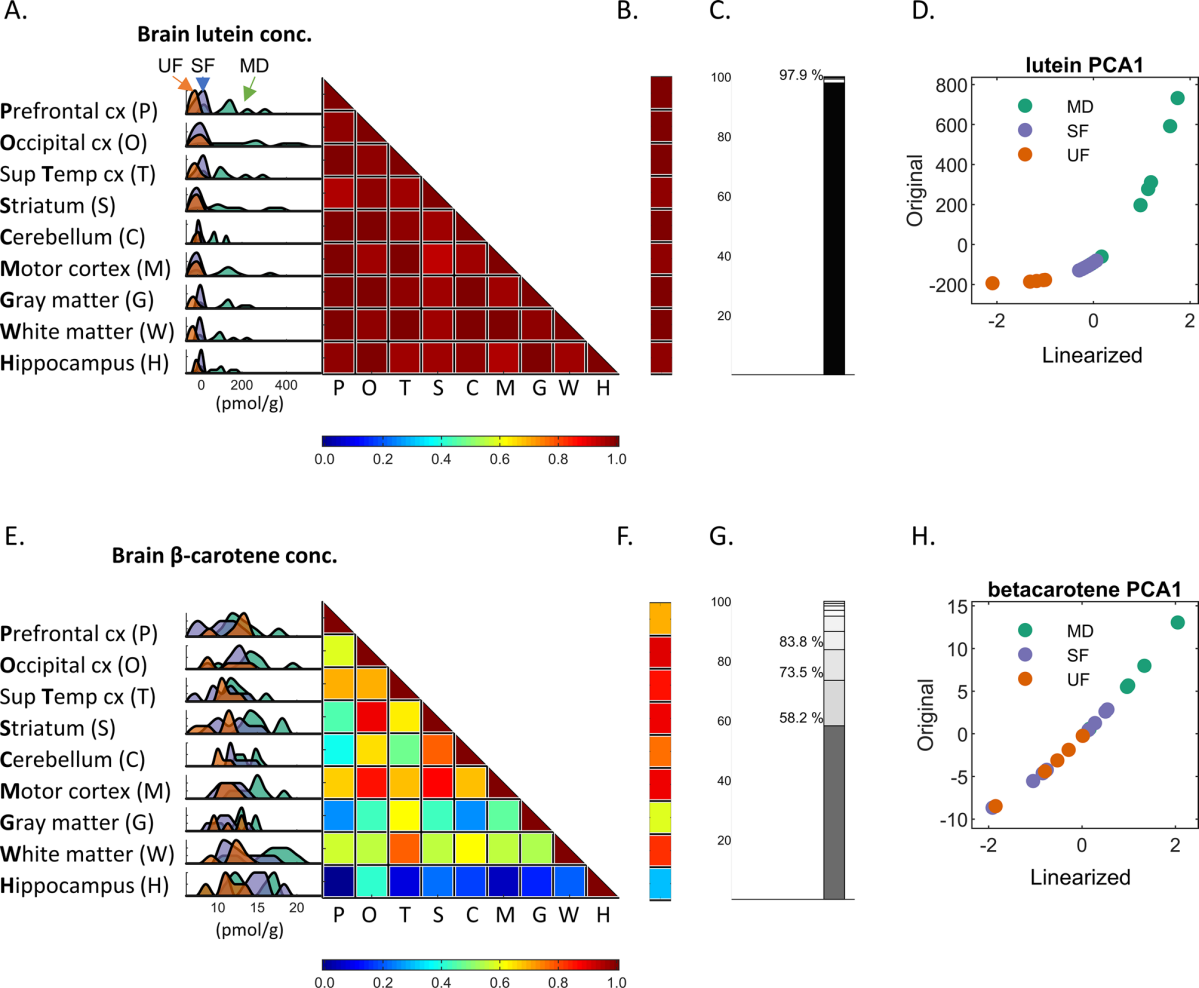
Carotenoid concentrations and composite index. (**A**) Correlation of lutein concentrations across brain areas with color-coded numerical scale and a companion insert displaying histograms with the concentration of lutein in each brain area, color-coded by group: MD, mixed-diet (green); UF, unsupplemented formula (blue); SF, supplemented formula (orange). (**B**) Correlation of the first PCA component with the concentration of lutein for each brain area using the same color-coded scale used in (**A**). (**C**) Explained variance for each component after orthogonalization of lutein’s concentration across brain areas using PCA. (**D**) PCA’s first component row-scores (y-axis) and transformed scores using a logarithmic (BoxCox) transformation. Similar data for β-carotene is shown in (**E**–**H**).

Lutein concentrations in the nine brain areas studied (prefrontal cortex, occipital cortex, superior temporal cortex, striatum, cerebellum, motor cortex, isolated frontal gray matter, frontal white matter, and hippocampus) were highly correlated. The same was true of β-carotene in the nine brain areas. The median Pearson correlation value (interquartile range) was 0.98 (0.97–0.99) for lutein and 0.56 (0.32–0.67) for β-carotene, as shown in Fig. 3A,E. Therefore, for each carotenoid we calculated a global concentration index using PCA. The first PCA component accounted for 97.9% and 58.2% of the observed variance for lutein and β-carotene, respectively (Fig. 3C,G). The first component was also highly correlated with the concentration of the corresponding carotenoid for each brain area (from 0.980 to 0.997 for lutein and 0.31 to 0.90 for β-carotene, as also shown in Fig. 3B,F), suggesting that the first PCA component can be used effectively as a surrogate for carotenoid concentration. In preparation for PLSR models, PCA first components were normalized using a logarithmic transformation, as shown in Fig. 3D,H.

### Associations between functional connectivity and brain concentration of carotenoids

**Figure 4:**
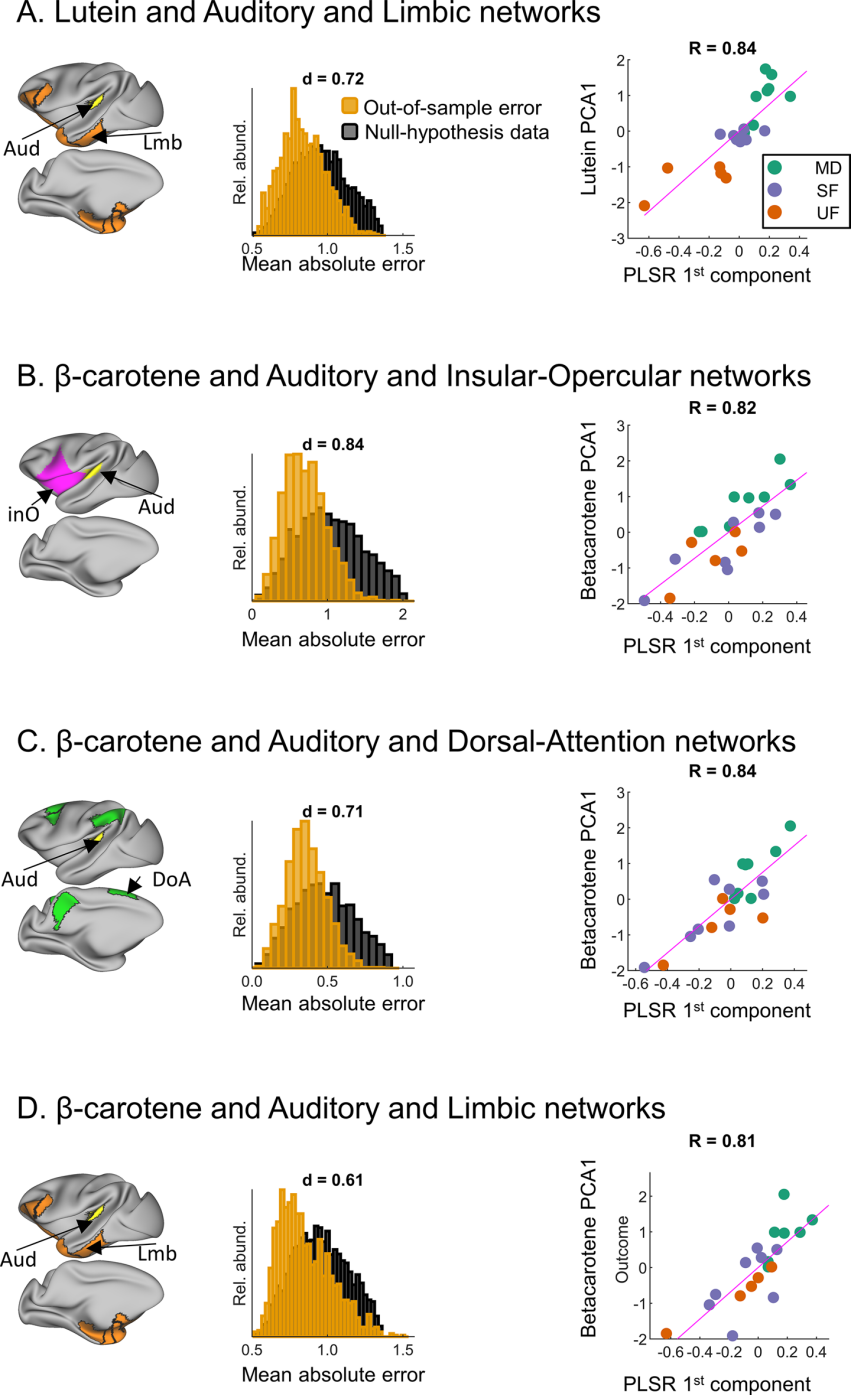
Associations between functional connectivity and carotenoid brain concentration. (**A**) Lutein brain concentration (first PLSR component) was associated with functional connectivity between the Auditory and Limbic networks (see Fig. 1 for network color codes). (**B**–**D. **β-carotene brain concentration (first PLSR component) was associated with functional connectivity between (**B**). Auditory and Insular-Opercular; (**C**) Auditory and Dorsal Attention; and (**D**) Auditory and Limbic networks. Column 1 indicates the location of each functional network pair (see Fig. 1 for color codes) in a highly inflated projection of the brain cortex with left-lateral and left-medial views. Column 2 shows the distribution of the out-of-sample mean absolute errors (n = 1330, yellow), against null-hypothesis data (n = 4000, black). Column 3 is a scatter plot displaying, for each subject, the predicted outcome and the first PLSR component, color-coded by group: MD, breastfed (green); UF, un-supplemented formula (blue); SF, supplemented formula (orange).

Associations between functional connectivity and carotenoids' concentration were estimated using the data closest in time of tissue collection, i.e., using the brain scan acquired at 6 mo. We found that functional connectivity was significantly associated with brain concentration of lutein and β-carotene, as accounted by PLSR models. For lutein, only models trained with connectivity data for the Auditory and Limbic network pair predicted out-of-sample data (i.e., normalized first PCA of lutein’s concentration) with an effect size > 0.5 (*Cohen’s d* = 0.72, predicting out-of-sample scores *versus* predicting null data). In other words, the mean absolute error between the predicted and the composite index of actual concentrations was significantly smaller than the randomized permuted data (null-hypothesis). Predicted values and the first PLSR component were also highly correlated ($R=0.84$), as shown in Fig. 4A. For β-carotene, three functional network pairs predicted out-of-sample data with an effect size > 0.5. Models trained with connectivity data from the Auditory and Insular-Opercular network pair predicted out-of-sample composite index values with a large effect size (*Cohen’s d* = 0.84), as shown in Fig. 4B. Models trained with data from the Auditory and Dorsal Attention and Auditory and Limbic pairs predicted out-of-sample outcome with a medium effect size (*Cohen’s* d = 0.71 and $0.61$, respectively), as shown in Fig. 4C,D. Predicted values and the first PLSR components were also highly correlated ($0.82$, $0.84$ and $0.81$ for Aud and InO, Aud and DoA, and Aud and Lmb, respectively), as also shown in Fig. 4B–D.

## Discussion

We aimed to identify differences in brain maturation using functional connectivity in a well-controlled diet intervention protocol using a highly translational macaque model. We have previously reported that this intervention led to differential concentrations of lutein on different brain areas. β-carotene, in contrast, only showed a difference across groups in the cerebellum. This follow-up study aimed to identify differences in brain maturation patterns among groups. Our observations generally indicated that by 6 months of age the UF formula resulted in a larger divergence from the pattern of functional connectivity observed in the MD group, a naturally fed group of macaque infants. The UF formula was not supplemented with lutein/carotenoids so that carotenoid intakes were markedly lower than that of the other groups, as indicated in Table 1. In contrast, the SF formula was supplemented with lutein and other carotenoids, and its 6-month pattern of functional connectivity was more like that of the MD infants. The functional connectivity patterns of the formula groups were different for the somatomotor and the somatomotor-visual networks implicating carotenoid supplementation in the development of those networks. In addition, the SF group had a maturation pattern similar to the pattern observed for the MD group in the auditory and dorsal attention network pair and the auditory and limbic network pair, which was not the case for the UF group.

Interestingly, the UF and SF groups showed similar patterns at 2- and 4-months. The SF group was different from the MD group at 2 months of age for insular opercular-somatomotor connectivity; however, this difference disappeared at 4 and 6 months. At 4 months of age, the MD group had positive connectivity in the dorsal attention-somatomotor network pair, whereas both formula groups had negative connectivity, and were not different from each other. Overall, we found that supplementation may take some time to take effect, since by 6-mo of age the SF group showed more similar patterns to the MD group, differing from the UF group. This finding indicated supplementation of formula with carotenoids influenced the development of these brain networks. Previous results from these monkeys^20^ implicate lutein as being an important carotenoid in brain maturation since its concentration in the infant’s brains was dramatically higher than those of the other carotenoids. To understand the relevance of our network observations, we compared the formula-fed groups to the MD group through post hoc comparisons.

It is challenging to disentangle the combined effect of nutrition and rearing conditions on brain maturation when comparing laboratory reared formula-fed infants to infants that were breastfed by their dam. Differences in rearing of non-human primates is known to have significant impact on social behavior, social cognition and affective behavior, as well as brain development^67–69^. With this in mind, we conducted a post hoc analysis to assess the directional context of the differences detected on network maturation between the formula fed groups. The post hoc analysis revealed a distinct pattern for both the somatomotor and the somatomotor-visual network pairs at 6 months of age. In both cases, the UF group was significantly different from the MD group, but the SF group was not. The post hoc analysis also revealed additional, less distinct 6-month connectivity effects in the auditory-dorsal attention and auditory-limbic network pairs. In both cases, connectivity in UF infants was different from that in the MD group. Notably, for those two functional pairs the only significant difference in connectivity values were for the UF versus MD groups. Taken together, these findings suggest that lack of supplementation led to a divergent maturation pattern in comparison to the MD group. These observations generally indicated that by 6 months of age the UF formula resulted in a larger divergence from the pattern of functional connectivity observed in the MD group in the somatomotor, somotomotor and visual, auditory and dorsal attention and auditory and limbic systems. We consider this to be an important observation since the SF formula was supplemented with nutrient compounds not added to most commercially-marketed infant formulas; most are not supplemented with lutein or the other carotenoids. While unsupplemented formulas have some baseline carotenoid content, such concentration levels might not be sufficient.

Non-human primates offer a unique translational opportunity to study the impact of nutrition on brain development. They, like humans, deposit lutein in the brain and retina^20^. In addition, non-human primates have high cognitive capacity, exhibit social behavior and have cerebral cortical areas and networks similar to those found in the human brain^42–44^. This allows the controlled study of early dietary interventions on functional cortical organization later in life. For example, a prior study in macaques showed that dietary omega-3 fatty acids may increase functional connectivity in early visual pathways and may have an impact on large-scale functional network organization, including the dorsal attention, and cingulo-opercular networks^70^. Similarly, here we found differences between the UF and the MD group within the Auditory and Dorsal Attention networks.

Our observations of connectivity differences within the somatomotor network pairs are consistent with human research which supports positive effects of lutein on the performance of tasks requiring visual-motor and vestibular-motor coordination. Renzi et al.^71^ found that macular pigment optical density, a measure of lutein and zeaxanthin concentration, was positively correlated with reaction time and balance in adults. These processes are supported by the integration of the motor cortex with visual stimuli (visual cortex) and vestibular function (insular cortex). Motor cortex lutein concentration is implicated as a causative factor by these findings since macular pigment optical density is highly correlated with lutein concentrations in the motor cortex^32,35,72^. Additionally, in human infants, the development of motor skills was associated with transitions from disperse to focal brain connectivity patterns at the ages of 6 to 12 months^73^. Our findings suggest that a diet lacking lutein might lead to atypical maturation patterns of functional connectivity of the somatomotor network with other networks. This may be important for the development of a variety of motor cortex dependent behavioral responses beyond simple components of movements, including defensive movements, reaching and hand-to-mouth movements, as reported in primate research^74^. Additional research is necessary to test this possibility.

Previous research revealed that concentrations of lutein across nine brain areas in these infants were significantly increased by carotenoid supplementation, but the MD group had several-fold higher concentrations compared to the SF group^20^. Levels of β-carotene did not differ between the formula groups and were only slightly higher in some regions in the MD group^20^. Here, we quantified associations of network connectivity patterns with a global concentration index for each carotenoid. Brain levels of both lutein and β-carotene were positively correlated with connectivity in the auditory-limbic network pair, while β-carotene was correlated with connectivity of the auditory network with the insular-opercular and dorsal-attention networks, both key networks involved in cognitive function. It is not known why these carotenoids would have selective effects on the connectivity of the auditory cortex with both the limbic system and higher-order cortical regions, but this effect calls for further study. In this regard, it is relevant to mention that there are reports linking impaired connectivity between the auditory and limbic networks with tinnitus. Tinnitus is the sensation of hearing noises without a real external auditory signal and might be related to oxidative stress. One of the hypotheses supported by neuroimaging findings^75–77^ is that tinnitus emerges after the inability of the limbic system to suppress a “tinnitus” signal caused by degeneration of the auditory cortex^78^. While our findings cannot in any way suggest a causal mechanism between tinnitus and carotene supplementation, they do indicate the networks whose functional connectivity is highly correlated with carotene concentration might be more susceptible to oxidative stress caused by carotene deficiencies.

Functional MRI offers a unique opportunity to non-invasively characterize functional brain development and can also be used to bridge findings across species since some human-defined brain systems are preserved in non-human primates and even rodents^79,80^. This modality utilizes the blood-oxygenation-level-dependent (BOLD) signal, which measures changes in the magnetic properties of oxygenated and de-oxygenated hemoglobin in the brain’s blood^81^. This signal combines non-linearly many complex physiological processes. However, if there is a local change in neural activity, that brain area will also exhibit a change in signal intensity that can be detected by the MRI scanner, even if the participant performs no activity when the data is acquired^82^. Patterns of brain connectivity might support behavioral differences related to higher order processes^8^ such as executive function, learning and memory or self-regulation^83,84^, to name a few. In this study we found significant differences in connectivity patterns for the 3-way interaction of diet, age, and networks. As described before, those changes landed not only in the visual cortex but also in systems that in humans support cognition. Since the concentration of carotenoids was predicted by connectivity patterns in those systems, we believe the observed differences in maturation patterns are directly related to differences in diet. It is important to mention, however, that this study did not aim to characterize behavioral differences related to differences in diet.

Functional MRI, however, has inherent challenges driven by both signal to noise ratio, head-movement and the need to properly align scans from different subjects. We used validated methods to overcome these methodological challenges and the relatively small number of infant macaques used in this study. Specifically, we used surface-based registration^85^ and connectotyping^45^. Surface-based registration can improve brain alignment, the signal-to-noise ratio and cut out confounding signal from white matter, cerebrospinal fluid, and bone. Additionally, it helps to avoid overlap in areas of cortical folding, where regions containing different functional information may be close in Euclidian distance in volume space, but further away from another on the cortical sheet. In addition, by using connectotyping we can more accurately estimate patterns of brain connectivity using limited data^45,86^.

This study has several limitations, including the inability to isolate the contributions of individual carotenoids and the relatively short duration of the study. We acknowledge that the ideal design would have required more than three groups in order to test the individual effects and their interactions. Given the challenges of a large per group sample in this longitudinal macaque study, we decided to combine carotenoid/lutein supplementation and different sources of α-tocopherol. As previously reported^46^, there were no differences in the brain concentration of total α-tocopherol among groups for the nine brain regions assessed^46^. In contrast, brain lutein concentration was dramatically higher than that of β-carotene in the MD and SF groups, but not the UF group^20^ and no lycopene was found in any of the nine brain regions tested^20^. Thus, we believe that higher brain lutein is the most likely driving force behind the observed changes in brain maturation. However, potential interactions between lutein, β-carotene and source of α-tocopherol cannot be ruled out. It is of note that the α-tocopherol natural and synthetic stereoisomer profiles in the brains of the MD and SF infants were markedly different, while the UF group was intermediate^42^. This pattern suggests that α-tocopherol source was not important in the network pairs described here. Another limitation corresponds to the small sample sizes inherent to most nonhuman primate developmental studies. However, the differences between the formula groups are clear and not diminished by the sample size constraint. While, in general, large samples are required to derive strong conclusions about brain behavior associations^87^, there are alternatives that are as effective as having large samples, including the design of hypothesis-driven interventions, the use of longitudinal data and using methods to improve the signal-to-noise ratio of the variables of interest^88^. This study used a well-designed intervention based on diet primarily. We also used longitudinal data acquired at 2, 4 and 6 months of age and we used several procedures to maximize the data signal to noise ratio, including surface-based registration, connectotyping and a rigorous QC based on structural and functional data, where we excluded data from scans with imperfect delineation of gray and white matter or insufficient data for functional connectivity analysis. In addition, we used a stringent repeated measures ANOVA to characterize any potential difference among the variables of interest. This positive finding was followed up by post-hoc stats to characterize the cases driving the differences. However, we acknowledge that while data was acquired in a critical developmental period (2–6 months of age) where the most dramatic changes in brain structure and function occur, a larger sample size would improve the detection of smaller effects. Despite these limitations, repeated measures ANOVA approach was able to identify significant differences in functional connectivity within specific brain networks.

Previous reports in these infants indicated that white and gray matter development measured by fractional anisotropy was greater in the MD group compared with both formula groups^47^. Here, we found that network connectivity of Somatomotor, Somatomotor-Visual, Auditory-Dorsal Attention, and Auditory-Limbic networks in the SF group more closely resembled the MD group when compared to the UF group. Results of this longitudinal study suggest that supplementation may take some time to take effect, since by 6-mo of age the SF group showed more similar patterns to the MD group, differing from the UF group. These findings implicate a role for lutein, and possibly β-carotene, in the development of these network pairs. Support for this conclusion was provided by associations between brain concentrations of lutein and β-carotene and connectivity between the auditory network and networks subserving emotion regulation and cognitive function. In conclusion, these findings suggest that variation in carotenoid intake are related to aspects of functional brain development. Importantly, a formula with lower carotenoid levels resulted in the largest divergence from breastfeeding in functional connectivity.

## Supplementary Information

Supplementary Information.

## Data Availability

The datasets generated during and/or analyzed during the current study are available from the corresponding author on reasonable request.
