# Supplementary material for: Carotenoids improve the development of cerebral cortical networks in formula-fed infant macaques
Source: Sci Rep. 2022 Sep 8;12:15220. doi: 10.1038/s41598-022-19279-1 (PMC9458723; doi:10.1038/s41598-022-19279-1)
Supplement: Supplementary file 1 — Supplementary Information. [file 41598_2022_19279_MOESM1_ESM.pdf]

Imaging acquisition and protocol

MRI scans were acquired at 2, 4 and 6 months of age. Animals were sedated with 5 mg/kg of ketamine and intubated, and anesthesia was maintained with 1% isoflurane vaporized in 100% oxygen. Vital signs were monitored continuously and homeostasis was maintained. Four T1-weighted (TR=2500ms, TE=3.86ms; 0.5 mm iso-voxels, 128 slices, FOV=108 x 128 mm) and one T2-weighted (TR=10640ms, TE1/TE2=11/95ms; echo train length = 8, slice thickness = 1 mm, in-plane nominal resolution: 0.5 × 0.5 mm, sampling matrix: 256 × 256, and 60 slices) structural images were acquired for registration. We collected thirty minutes of blood-oxygen-level dependent (BOLD) contrast imaging data using a gradient echo-planar imaging (EPI) sequence (TR= 2070 ms, TE=25 ms, FA=90o, 1.5 mm iso-voxels, 32 slices with interleaved acquisition, FOV 96 x 96 mm). A field map scan data (TR = 450 ms, TE = 5.19 ms/7.65 ms, FA = 60°, 1.25 × 1.25 × 2 mm voxels, 40 slices, FOV) was also acquired to correct for image distortions in the BOLD signal.

MRI processing. Data was processed using surface-based registration following the standards and steps proposed by the Human Connectome Project (HCP) [1], with macaque specific modifications. The HCP pipeline includes the use of FSL [2–4] and Freesurfer [5–7]. To improve image registration, we incorporated tools from the Advanced Normalization Tools (ANTs) [8] version 1.9 (http://stnava.github.io/ANTs/) in combination with in-house established tools. Details of this implementation have been described previously by our group [9]. Briefly, T1 weighted scans were registered to the Yerkes19 macaque atlas [10] and averaged together to improve the signal to noise ratio. This original alignment was refined using boundary-based registration [11]. Then, optimally aligned T1-weighted images were segmented using recon-all from FreeSurfer. Cortical thickness was calculated in native space (i.e., subject-specific) at a spatial density of 0.9 mm intervertex distance. These values then were downsampled and reported at a spatial resolution that has an intervertex spacing of 2 mm. This space is termed "the standard grayordinate space" and has of 91,282 anchor points (grayordinates) in the gray matter (surface and subcortical ROIs) [1].

EPI data was registered, processed and reported at each grayordinate. The BOLD data was corrected for field distortions and processed by doing a preliminary 6 degrees of freedom linear registration to the first frame. After this initial alignment, the average frame was calculated and used as final reference. Next, the BOLD data was registered to this final reference and to the T1-weighted volume, all in one single step, by concatenating all the individual registrations into a single transformation matrix. The additional preprocessing consisted of correcting for the effect of signals known to obscure the estimates of functional connectivity [12], including activity from the whole brain, ventricles, gray matter, head displacement and rotation and their squares and derivatives [13,14]. Finally, timecourses were filtered using a first order Butterworth band pass filter with cutting frequencies between 9 and 80 *mHz*.

Quality checks. All the data were visually inspected to ensure that they were processed and registered properly. Visual inspection verified that no artifacts remained, brain was fully acquired and processed and that the gray matter was accurately delineated.

Motion censoring. We used only BOLD data from low head-movement frames [15], and BOLD data with a frame displacement higher than 0.4 mm were discarded. Removed frames were selected based on their total relative movement in any direction and their proximity to discarded frames [16], and segments with less than five continuous frames were discarded. In this study we only kept participants with at least 5 minutes of surviving frames (*i.e.* 144 frames), and

connectivity matrices were calculated using the same number of frames, sampled randomly out of the surviving frames.

Delineation of regions of interests (ROIs) and functional networks. Resulting BOLD data from each grayordinate were parcellated using a predefined macaque cortical parcellation schema [17] consisting of 82 regions of interest (ROIs) that cover the entire cortex, as shown in Figure 1. These 82 ROIs were then grouped into the 7 following functional networks, as defined in Grayson et al., 2016, with the abbreviation and the number of component ROIs for each network shown in parentheses: auditory (Aud, n=4), default mode (Def, n=26), dorsal attention (DoA, n=10), insular-opercular (InO, n=9), limbic (Lmb, n=12), somatomotor (SoM, n=13), and visual (Vis, n=8). These 7 networks are illustrated in Figure 1, panel A.

Functional connectivity. Functional connectivity was characterized using connectotyping [19] using parcellated data [17]. In connectotyping, functional connectivity is obtained by linear models that relate BOLD data among ROIs, not by paired correlation between ROIs. Linear models are fit to predict the BOLD signal of each ROI as a function of the weighted contribution of the BOLD data from all the other ROIs (see [19] for details). Briefly, for each participant, autocorrelation was removed first from each ROI's time courses using five auto-regressive terms. Head-movement mask was applied on the pre-whitened time courses (i.e., after removal of autocorrelations) to select only low-head-movement data. To make sure all the connectivity matrices were calculated using the same amount of data (5 minutes), for each participant we selected the same number of frames (n=144) from the low head-movement data. Those 144 frames were selected randomly to avoid bias with regard to head movement. Next, for each ROI, a model was fit to predict time courses based on the linear combination of all the other ROIs via truncated singular value decomposition (tsvd). The optimal number of preserved components was selected using cross-validation. To do this, the data from each ROI were randomly partitioned into two datasets, modeling and prediction, respectively having 70 and 30% of the available frames. Linear models trained using 70% of the data were tested on the other partition and the number of components with the largest out-of-sample accuracy were recorded per ROI. This procedure was repeated 10 times and the optimal number of components was determined by the average across runs. The resulting number of components was used to calculate the connectivity matrices (i.e., connectotypes) using all the low-head movement frames. This procedure was done independently for each participant. Supplementary Figure 1 shows the mean connectivity values per group and age. The result was a directed connectivity matrix for each scan with 6,642 connections ($6{,}642 = 82 \times (82 - 1)$). Resulting directed matrices have relatively similar connectivity values for each connection pair. In other words, for the ROIs A and B, the connectivity value from A to B is similar to the connectivity value from B to A. Hence, we decided to calculate the average between those values. This operation was implemented by symmetrizing each matrix ($S = (M + M')/2$, where M is a connectivity matrix, M' is the transpose of the matrix M, and S is the resulting symmetrized matrix) and the resulting matrices had 3,321 unique connectivity values. Resulting connections were assigned to the functional network pair each constituent ROI belongs to. In more detail, since a connection is defined by two ROIs and each ROI belongs to a unique functional network [17], as shown in Figure 1, connections were assigned to the unique network pair defined by the network each ROI belongs to (n=28). Count of connections per network pair are reported in the Supplementary Table 1.

Statistical analysis

In this study we aimed to identify differences in functional connectivity among groups (MD, SF and UF) with different nutritional histories and ages. Differences in functional connectivity were assessed using a 3-way repeated measures ANOVA test using all the connectivity values *per*

participant, for the factors group (3 levels: MD, SF and UF), age (3 levels: 2, 4, and 6mo) and networks (28 levels: Aud-Aud, Aud-Def, …, which correspond to each functional network pair). See details below in the subsection "Grouping connectivity values as a function of within- and between-subjects factors for functional connectivity values" For positive effects, post-hoc analyses consisted of comparing connectivity values between groups after removal of the effect of repeated measures using the function multcompare in Matlab.

As a secondary analysis, we set to determine if the concentration values of lutein and beta-carotene in the brain are associated with functional connectivity using multivariate statistics [20–23]. We decided to use partial least squares regression (PLSR) [24] to fit models to predict carotene's brain concentration as a function of functional connectivity, one model for each functional network pair. If an association exists for a functional network pair, the model should be able to predict carotene's concentration beyond chance. Risk of overfitting was reduced by using regularization and significance of the associations was determined by comparing out-of-sample performance using hold-cross association versus null data. Concentration of carotenoids were measured from nine different brain locations [25]. Concentration values, however, were highly correlated among brain areas. Hence, we calculated two global indices, one for lutein and another one for beta carotene using principal component analysis (PCA). As the first component explained most of the observed variance on each case, we used the first component as predicted variable. Notice that we did not attempt to determine causality but rather significant associations. For this reason, it did not matter which variable (carotene's concentration or functional connectivity) was used as dependent or independent variable. Using functional connectivity as independent variable was a better choice algorithmically. Doing the opposite would require calculation of massive univariate models to relate the same carotene's concentration to each unique connectivity value per participant, implying that carotene's concentration (a single value per participant) was able to predict all the variability measured by functional connectivity, which, given our sample size, would be prone to overfitting.

Details of each step are presented in the following subsections.

Grouping connectivity values as a function of within- and between-subjects factors for functional connectivity values. Each participant (n=21, 8 MD, 8 SF, and 5 UF) had 3 scans (acquired at 2, 4 and 6 mo) and 3,321 unique connectivity values per scan. Hence, there were 9,963 connectivity values per participant. Those 9,963 connectivity values were repeated measures since they came from the same participant and belonged to two distinct within-subjects factors (repeated factors): functional network pair (Aud-Aud, Aud-Def, …) and age (2m, 4m, and 6m). Furthermore, those connectivity values had group (MD, SF or UF) as a between-subjects factor.

Repeated measures ANOVA to identify differences in functional connectivity among groups. We used a 3-way repeated measures ANOVA test in Matlab [20,22,26] to characterize differences in functional connectivity among groups (MD, SF, and UF), the repeated factors age (2m, 4m, 6m), functional network pair (Aud-Aud, Aud-Def, …) and their interactions [20,26]. The test was done in two steps. First, a linear mixed effects model was fit to predict functional connectivity values as a function of group (i.e., MD, SF and UF) using age and functional network pair as within-subject factors. Next, corrected values were grouped based on between- and within-subject factors, and then an ANOVA test was used to characterize differences among factors. Mauchly's test of sphericity was used to test for differences in variance among the groups being compared, and p-values were adjusted accordingly using the correction factor

epsilon. Epsilon-adjusted p-values were corrected for multiple comparisons using the Tukey–Kramer method, and 0.05 was used as threshold for significance.

Post-hoc analysis to identify the factors driving the observed differences. As post-hoc analysis aimed to identify the functional network pair(s) with differences in functional connectivity, we compared marginal means for each factor found to be significant in the main repeated measures ANOVA. As mentioned before, Epsilon-adjusted p-values were corrected for multiple comparisons using the Tukey–Kramer method, and 0.05 was used as threshold for significance.

Correlation of functional connectivity measures with brain lutein and beta-carotene concentrations. Given the high correlation that the concentration of both lutein and beta-carotene have across the nine brain regions that were analyzed (dorsolateral prefrontal cortex, occipital cortex, superior temporal cortex, striatum, cerebellum, mortor cortex, isolated frontal gray matter, frontal white matter, and hippocampus), we used PCA to calculate a global index for each carotenoid. The first PCA component was used as global index of carotenoid concentration. PCA scores were normalized using a logarithmic transformation (Boxcox transformation) [27], where the logarithmic base was optimized by gradient descent. Associations between lutein and beta-carotene indices (PCA scores) and functional connectivity were assessed using partial least squares regression (PLSR) models [24] using imaging data from the last scan (i.e. at 6mo) which is the time just before samples were taken to measure lutein's and beta-carotene's concentrations. For each functional network pair (N=28: Aud-Aud, Aud-Def, …) we used PLSR models to characterize associations between lutein and betacarotene indices (PCA scores) and functional connectivity. PLSR models relate predicted and predictor variables after transforming them into a new set of latent variables (or components) that maximizes outcome prediction [24].. A tuning parameter is the number of components (i.e. dimensions) used to project the data on. Increasing the number of components tends to increase within-sample accuracy but results might not generalize. Here we decided to use 4, a relatively low number of components in all the models, to decrease the likelihood of overfitting.

For each case (i.e., each combination of functional network pair and lutein and beta-carotene index), three participants were reserved to test the accuracy of the predictions (partition validation) while the remaining participants were used to train models (partition modeling). Data from the partition modeling were used to calculate PLSR models using sequentially all the possible number of components. Resulting models were used to predict scores in the partition validation. Subsampling (i.e., partitioning the data in the two different partitions: modeling and validation) was repeated until exploring all the possible combinations that can be done reserving three participants for validation. This corresponds to 1,330 unique cases, since those are all the unique combinations of subsamples that can be done given samples of 21 surviving participants, reserving 3 participants for validation. Model accuracy was estimated by calculating the mean absolute error of the predictions. Performance of the models versus chance was compared by also training models using null data for each functional network pair. Null data were created by permuting randomly 4,000 times the assignments between connectivity and lutein and beta-carotene indices within the data used for modeling. Models trained with permuted data were also tested with out-of-sample un-permuted data. Significance was determined based on the comparison of the distribution of the mean absolute errors against predicting null-hypothesis data. This comparison was quantified using Cohen's effect size. Here we report results when predictions had at least a medium effect size ($Cohen's\ d > 0.5$). We calculated the correlation coefficient between the first component of each PLSR model and the predicted outcome (i.e., composite indices of lutein and beta-carotene concentrations) to estimate associations between imaging and concentration of carotenoids in the brain. Each

PLSR component corresponds to the orthogonalization of all the unique connectivity values such that outcome prediction is maximized. The first component explained most of the variance of the data.

References

1. Glasser, M. F. *et al.* The minimal preprocessing pipelines for the Human Connectome Project. *Neuroimage* **80**, 105–24 (2013).
2. Smith, S. M. *et al.* Advances in functional and structural MR image analysis and implementation as FSL. *Neuroimage* **23**, S208--19 (2004).
3. Jenkinson, M., Beckmann, C. F., Behrens, T. E. J., Woolrich, M. W. & Smith, S. M. FSL. *Neuroimage* **62**, 782–790 (2012).
4. Woolrich, M. W. *et al.* Bayesian analysis of neuroimaging data in FSL. *Neuroimage* **45**, S173--86 (2009).
5. Dale, A. M., Fischl, B. & Sereno, M. I. Cortical surface-based analysis. I. Segmentation and surface reconstruction. *Neuroimage* **9**, 179–194 (1999).
6. Desikan, R. S. *et al.* An automated labeling system for subdividing the human cerebral cortex on MRI scans into gyral based regions of interest. *Neuroimage* **31**, 968–980 (2006).
7. Fischl, B. & Dale, A. M. Measuring the thickness of the human cerebral cortex from magnetic resonance images. *Proc Natl Acad Sci U S A* **97**, 11050–11055 (2000).
8. Avants, B. B. *et al.* A reproducible evaluation of ANTs similarity metric performance in brain image registration. *Neuroimage* **54**, 2033–44 (2011).
9. Ramirez, J. S. B. *et al.* Maternal Interleukin-6 Is Associated With Macaque Offspring Amygdala Development and Behavior. *Cereb. Cortex* (2019) doi:10.1093/cercor/bhz188.
10. Donahue, C. J. *et al.* Using Diffusion Tractography to Predict Cortical Connection Strength and Distance: A Quantitative Comparison with Tracers in the Monkey. *J. Neurosci.* **36**, 6758–70 (2016).
11. Greve, D. N. & Fischl, B. Accurate and robust brain image alignment using boundary-based registration. *Neuroimage* **48**, 63–72 (2009).
12. Ciric, R. *et al.* Benchmarking of participant-level confound regression strategies for the control of motion artifact in studies of functional connectivity. *Neuroimage* **154**, 174–187 (2017).
13. Power, J. *et al.* Methods to detect, characterize, and remove motion artifact in resting state fMRI. *Neuroimage* (2013) doi:10.1016/j.neuroimage.2013.08.048.
14. Friston, K. J., Mechelli, a, Turner, R. & Price, C. J. Nonlinear responses in fMRI: the Balloon model, Volterra kernels, and other hemodynamics. *Neuroimage* **12**, 466–477 (2000).
15. Power, J. D., Barnes, K. A., Snyder, A. Z., Schlaggar, B. L. & Petersen, S. E. Spurious but systematic correlations in functional connectivity MRI networks arise from subject motion. *Neuroimage* **59**, 2142–2154 (2012).
16. Power, J. D. J. D. *et al.* Methods to detect, characterize, and remove motion artifact in resting state fMRI. *Neuroimage* **84**, 320–41 (2014).
17. Bezgin, G., Vakorin, V. A., van Opstal, A. J., McIntosh, A. R. & Bakker, R. Hundreds of brain maps in one atlas: registering coordinate-independent primate neuro-anatomical data to a standard brain. *Neuroimage* **62**, 67–76 (2012).
18. Grayson, D. S. *et al.* The Rhesus Monkey Connectome Predicts Disrupted Functional Networks Resulting from Pharmacogenetic Inactivation of the Amygdala. *Neuron* **91**, 453–66 (2016).
19. Miranda-Dominguez, O. *et al.* Connectotyping: Model Based Fingerprinting of the Functional Connectome. *PLoS One* **9**, e111048 (2014).

20. Rudolph, M. D. *et al.* Maternal IL-6 during pregnancy can be estimated from newborn brain connectivity and predicts future working memory in offspring. *Nat. Neurosci.* **21**, 765–772 (2018).
21. Rudolph, M. D. *et al.* At risk of being risky: The relationship between “brain age” under emotional states and risk preference. *Dev. Cogn. Neurosci.* **24**, 93–106 (2017).
22. Miranda-Domínguez, Ó. *et al.* Lateralized Connectivity between Globus Pallidus and Motor Cortex is Associated with Freezing of Gait in Parkinson’s Disease. *Neuroscience* **443**, 44–58 (2020).
23. Silva-Batista, C. *et al.* Cortical thickness as predictor of response to exercise in people with Parkinson’s disease. *Hum. Brain Mapp.* **42**, 139–153 (2021).
24. Rosipal, R., Kr, N., Krämer, N., Kr, N. & Krämer, N. Overview and recent advances in partial least squares. *Int. Stat. Optim. Perspect. Work. Subspace, Latent Struct. Featur. Sel.* 34–51 (2005).
25. Jeon, S. *et al.* Lutein Is Differentially Deposited across Brain Regions following Formula or Breast Feeding of Infant Rhesus Macaques. *J. Nutr.* **148**, 31–39 (2018).
26. Kovacs-Balint, Z. *et al.* Early Developmental Trajectories of Functional Connectivity Along the Visual Pathways in Rhesus Monkeys. *Cereb. Cortex* **29**, 3514–3526 (2019).
27. Montgomery, D. C. *Design and Analysis of Experiments, Sixth Edition*. vol. 37 (John Wiley & Sons, Inc., 2005).

Table 1. Grouping of connections *per* functional system pair.

Count	Functional system pair	Number of connections
1	Aud and Aud	6
2	Aud and Def	104
3	Aud and DoA	40
4	Aud and InO	28
5	Aud and Lmb	56
6	Aud and SoM	52
7	Aud and Vis	32
8	Def and Def	325
9	Def and DoA	260
10	Def and InO	182
11	Def and Lmb	364
12	Def and SoM	338
13	Def and Vis	208
14	DoA and DoA	45
15	DoA and InO	70
16	DoA and Lmb	140
17	DoA and SoM	130
18	DoA and Vis	80
19	InO and InO	21
20	InO and Lmb	98
21	InO and SoM	91
22	InO and Vis	56
23	Lmb and Lmb	91
24	Lmb and SoM	182
25	Lmb and Vis	112
26	SoM and SoM	78
27	SoM and Vis	104
28	Vis and Vis	28
	Total	3321

Supplementary Table 2. Post-hoc analysis for the 3-way interaction of diet, age and networks.

Functional Network pair	Age	Group 1			Group 2			Comparison		
		Name	mean fconn	Std. error	Name	mean fconn	Std. error	Group 1- Group 2	StdErr	pValue
Aud and Aud	2m	MD	0.030	0.014	SF	0.050	0.014	-0.020	0.019	0.564
Aud and Aud	2m	SF	0.050	0.014	MD	0.030	0.014	0.020	0.019	0.564
Aud and Aud	2m	SF	0.050	0.014	UF	0.031	0.017	0.019	0.022	0.670
Aud and Aud	2m	UF	0.031	0.017	SF	0.050	0.014	-0.019	0.022	0.670
Aud and Aud	2m	MD	0.030	0.014	UF	0.031	0.017	-0.001	0.022	0.999
Aud and Aud	2m	UF	0.031	0.017	MD	0.030	0.014	0.001	0.022	0.999
Aud and Def	2m	MD	0.001	0.002	SF	-0.003	0.002	0.004	0.002	0.226
Aud and Def	2m	SF	-0.003	0.002	MD	0.001	0.002	-0.004	0.002	0.226
Aud and Def	2m	UF	-0.004	0.002	MD	0.001	0.002	-0.004	0.003	0.302
Aud and Def	2m	MD	0.001	0.002	UF	-0.004	0.002	0.004	0.003	0.302
Aud and Def	2m	SF	-0.003	0.002	UF	-0.004	0.002	0.000	0.003	1.000
Aud and Def	2m	UF	-0.004	0.002	SF	-0.003	0.002	-0.000	0.003	1.000
Aud and DoA	2m	MD	-0.002	0.005	UF	-0.018	0.006	0.016	0.008	0.121
Aud and DoA	2m	UF	-0.018	0.006	MD	-0.002	0.005	-0.016	0.008	0.121
Aud and DoA	2m	MD	-0.002	0.005	SF	-0.015	0.005	0.013	0.007	0.166
Aud and DoA	2m	SF	-0.015	0.005	MD	-0.002	0.005	-0.013	0.007	0.166
Aud and DoA	2m	SF	-0.015	0.005	UF	-0.018	0.006	0.003	0.008	0.911
Aud and DoA	2m	UF	-0.018	0.006	SF	-0.015	0.005	-0.003	0.008	0.911
Aud and InO	2m	UF	-0.007	0.007	SF	0.001	0.005	-0.009	0.008	0.561
Aud and InO	2m	SF	0.001	0.005	UF	-0.007	0.007	0.009	0.008	0.561
Aud and InO	2m	MD	-0.003	0.005	SF	0.001	0.005	-0.005	0.007	0.794
Aud and InO	2m	SF	0.001	0.005	MD	-0.003	0.005	0.005	0.007	0.794
Aud and InO	2m	UF	-0.007	0.007	MD	-0.003	0.005	-0.004	0.008	0.885
Aud and InO	2m	MD	-0.003	0.005	UF	-0.007	0.007	0.004	0.008	0.885
Aud and Lmb	2m	UF	-0.008	0.005	MD	-0.001	0.004	-0.007	0.006	0.538
Aud and Lmb	2m	MD	-0.001	0.004	UF	-0.008	0.005	0.007	0.006	0.538
Aud and Lmb	2m	MD	-0.001	0.004	SF	-0.006	0.004	0.005	0.005	0.664
Aud and Lmb	2m	SF	-0.006	0.004	MD	-0.001	0.004	-0.005	0.005	0.664
Aud and Lmb	2m	SF	-0.006	0.004	UF	-0.008	0.005	0.002	0.006	0.947
Aud and Lmb	2m	UF	-0.008	0.005	SF	-0.006	0.004	-0.002	0.006	0.947
Aud and SoM	2m	MD	0.007	0.003	UF	0.002	0.004	0.006	0.005	0.533
Aud and SoM	2m	UF	0.002	0.004	MD	0.007	0.003	-0.006	0.005	0.533
Aud and SoM	2m	MD	0.007	0.003	SF	0.004	0.003	0.004	0.004	0.702
Aud and SoM	2m	SF	0.004	0.003	MD	0.007	0.003	-0.004	0.004	0.702
Aud and SoM	2m	UF	0.002	0.004	SF	0.004	0.003	-0.002	0.005	0.925

Aud and SoM	2m	SF	0.004	0.003	UF	0.002	0.004	0.002	0.005	0.925
Aud and Vis	2m	MD	-0.005	0.002	SF	-0.001	0.002	-0.004	0.003	0.427
Aud and Vis	2m	SF	-0.001	0.002	MD	-0.005	0.002	0.004	0.003	0.427
Aud and Vis	2m	UF	-0.003	0.003	SF	-0.001	0.002	-0.003	0.004	0.757
Aud and Vis	2m	SF	-0.001	0.002	UF	-0.003	0.003	0.003	0.004	0.757
Aud and Vis	2m	MD	-0.005	0.002	UF	-0.003	0.003	-0.001	0.004	0.916
Aud and Vis	2m	UF	-0.003	0.003	MD	-0.005	0.002	0.001	0.004	0.916
Def and Def	2m	MD	0.004	0.002	UF	0.001	0.002	0.004	0.003	0.481
Def and Def	2m	UF	0.001	0.002	MD	0.004	0.002	-0.004	0.003	0.481
Def and Def	2m	MD	0.004	0.002	SF	0.002	0.002	0.002	0.003	0.643
Def and Def	2m	SF	0.002	0.002	MD	0.004	0.002	-0.002	0.003	0.643
Def and Def	2m	SF	0.002	0.002	UF	0.001	0.002	0.001	0.003	0.923
Def and Def	2m	UF	0.001	0.002	SF	0.002	0.002	-0.001	0.003	0.923
Def and DoA	2m	UF	-0.010	0.002	MD	-0.005	0.002	-0.005	0.003	0.184
Def and DoA	2m	MD	-0.005	0.002	UF	-0.010	0.002	0.005	0.003	0.184
Def and DoA	2m	SF	-0.006	0.002	UF	-0.010	0.002	0.004	0.003	0.258
Def and DoA	2m	UF	-0.010	0.002	SF	-0.006	0.002	-0.004	0.003	0.258
Def and DoA	2m	MD	-0.005	0.002	SF	-0.006	0.002	0.001	0.002	0.968
Def and DoA	2m	SF	-0.006	0.002	MD	-0.005	0.002	-0.001	0.002	0.968
Def and InO	2m	SF	-0.003	0.003	UF	-0.008	0.004	0.005	0.005	0.558
Def and InO	2m	UF	-0.008	0.004	SF	-0.003	0.003	-0.005	0.005	0.558
Def and InO	2m	MD	-0.006	0.003	SF	-0.003	0.003	-0.004	0.004	0.646
Def and InO	2m	SF	-0.003	0.003	MD	-0.006	0.003	0.004	0.004	0.646
Def and InO	2m	UF	-0.008	0.004	MD	-0.006	0.003	-0.001	0.005	0.965
Def and InO	2m	MD	-0.006	0.003	UF	-0.008	0.004	0.001	0.005	0.965
Def and Lmb	2m	SF	-0.002	0.002	UF	0.000	0.002	-0.002	0.002	0.617
Def and Lmb	2m	UF	0.000	0.002	SF	-0.002	0.002	0.002	0.002	0.617
Def and Lmb	2m	UF	0.000	0.002	MD	-0.001	0.002	0.002	0.002	0.804
Def and Lmb	2m	MD	-0.001	0.002	UF	0.000	0.002	-0.002	0.002	0.804
Def and Lmb	2m	MD	-0.001	0.002	SF	-0.002	0.002	0.001	0.002	0.931
Def and Lmb	2m	SF	-0.002	0.002	MD	-0.001	0.002	-0.001	0.002	0.931
Def and SoM	2m	MD	-0.009	0.002	UF	-0.006	0.002	-0.002	0.003	0.663
Def and SoM	2m	UF	-0.006	0.002	MD	-0.009	0.002	0.002	0.003	0.663
Def and SoM	2m	MD	-0.009	0.002	SF	-0.007	0.002	-0.002	0.002	0.782
Def and SoM	2m	SF	-0.007	0.002	MD	-0.009	0.002	0.002	0.002	0.782
Def and SoM	2m	SF	-0.007	0.002	UF	-0.006	0.002	-0.001	0.003	0.957
Def and SoM	2m	UF	-0.006	0.002	SF	-0.007	0.002	0.001	0.003	0.957
Def and Vis	2m	MD	-0.005	0.002	SF	-0.007	0.002	0.002	0.003	0.828
Def and Vis	2m	SF	-0.007	0.002	MD	-0.005	0.002	-0.002	0.003	0.828
Def and Vis	2m	MD	-0.005	0.002	UF	-0.006	0.003	0.001	0.004	0.956

Def and Vis	2m	UF	-0.006	0.003	MD	-0.005	0.002	-0.001	0.004	0.956
Def and Vis	2m	SF	-0.007	0.002	UF	-0.006	0.003	-0.001	0.004	0.971
Def and Vis	2m	UF	-0.006	0.003	SF	-0.007	0.002	0.001	0.004	0.971
DoA and DoA	2m	MD	0.003	0.005	SF	0.001	0.005	0.001	0.006	0.972
DoA and DoA	2m	SF	0.001	0.005	MD	0.003	0.005	-0.001	0.006	0.972
DoA and DoA	2m	MD	0.003	0.005	UF	0.001	0.006	0.001	0.007	0.982
DoA and DoA	2m	UF	0.001	0.006	MD	0.003	0.005	-0.001	0.007	0.982
DoA and DoA	2m	SF	0.001	0.005	UF	0.001	0.006	-0.000	0.007	1.000
DoA and DoA	2m	UF	0.001	0.006	SF	0.001	0.005	0.000	0.007	1.000
DoA and InO	2m	MD	-0.010	0.004	SF	-0.007	0.004	-0.004	0.006	0.814
DoA and InO	2m	SF	-0.007	0.004	MD	-0.010	0.004	0.004	0.006	0.814
DoA and InO	2m	UF	-0.009	0.005	SF	-0.007	0.004	-0.002	0.007	0.939
DoA and InO	2m	SF	-0.007	0.004	UF	-0.009	0.005	0.002	0.007	0.939
DoA and InO	2m	MD	-0.010	0.004	UF	-0.009	0.005	-0.001	0.007	0.978
DoA and InO	2m	UF	-0.009	0.005	MD	-0.010	0.004	0.001	0.007	0.978
DoA and Lmb	2m	MD	-0.011	0.002	UF	-0.004	0.003	-0.007	0.004	0.193
DoA and Lmb	2m	UF	-0.004	0.003	MD	-0.011	0.002	0.007	0.004	0.193
DoA and Lmb	2m	MD	-0.011	0.002	SF	-0.006	0.002	-0.005	0.003	0.254
DoA and Lmb	2m	SF	-0.006	0.002	MD	-0.011	0.002	0.005	0.003	0.254
DoA and Lmb	2m	UF	-0.004	0.003	SF	-0.006	0.002	0.001	0.004	0.927
DoA and Lmb	2m	SF	-0.006	0.002	UF	-0.004	0.003	-0.001	0.004	0.927
DoA and SoM	2m	MD	-0.003	0.003	UF	0.001	0.004	-0.004	0.005	0.609
DoA and SoM	2m	UF	0.001	0.004	MD	-0.003	0.003	0.004	0.005	0.609
DoA and SoM	2m	MD	-0.003	0.003	SF	-0.001	0.003	-0.002	0.004	0.861
DoA and SoM	2m	SF	-0.001	0.003	MD	-0.003	0.003	0.002	0.004	0.861
DoA and SoM	2m	SF	-0.001	0.003	UF	0.001	0.004	-0.002	0.005	0.871
DoA and SoM	2m	UF	0.001	0.004	SF	-0.001	0.003	0.002	0.005	0.871
DoA and Vis	2m	MD	-0.007	0.003	UF	-0.013	0.004	0.006	0.006	0.577
DoA and Vis	2m	UF	-0.013	0.004	MD	-0.007	0.003	-0.006	0.006	0.577
DoA and Vis	2m	MD	-0.007	0.003	SF	-0.011	0.003	0.003	0.005	0.770
DoA and Vis	2m	SF	-0.011	0.003	MD	-0.007	0.003	-0.003	0.005	0.770
DoA and Vis	2m	SF	-0.011	0.003	UF	-0.013	0.004	0.002	0.006	0.913
DoA and Vis	2m	UF	-0.013	0.004	SF	-0.011	0.003	-0.002	0.006	0.913
InO and InO	2m	MD	0.023	0.006	UF	0.009	0.007	0.014	0.009	0.293
InO and InO	2m	UF	0.009	0.007	MD	0.023	0.006	-0.014	0.009	0.293
InO and InO	2m	SF	0.021	0.006	UF	0.009	0.007	0.012	0.009	0.432
InO and InO	2m	UF	0.009	0.007	SF	0.021	0.006	-0.012	0.009	0.432
InO and InO	2m	MD	0.023	0.006	SF	0.021	0.006	0.003	0.008	0.945
InO and InO	2m	SF	0.021	0.006	MD	0.023	0.006	-0.003	0.008	0.945
InO and Lmb	2m	MD	0.002	0.002	UF	-0.002	0.003	0.004	0.003	0.454

InO and Lmb	2m	UF	-0.002	0.003	MD	0.002	0.002	-0.004	0.003	0.454
InO and Lmb	2m	MD	0.002	0.002	SF	-0.000	0.002	0.002	0.003	0.788
InO and Lmb	2m	SF	-0.000	0.002	MD	0.002	0.002	-0.002	0.003	0.788
InO and Lmb	2m	UF	-0.002	0.003	SF	-0.000	0.002	-0.002	0.003	0.798
InO and Lmb	2m	SF	-0.000	0.002	UF	-0.002	0.003	0.002	0.003	0.798
InO and SoM	2m	MD	-0.018	0.003	SF	-0.005	0.003	-0.013	0.004	**0.011**
InO and SoM	2m	SF	-0.005	0.003	MD	-0.018	0.003	0.013	0.004	**0.011**
InO and SoM	2m	MD	-0.018	0.003	UF	-0.007	0.003	-0.011	0.004	0.067
InO and SoM	2m	UF	-0.007	0.003	MD	-0.018	0.003	0.011	0.004	0.067
InO and SoM	2m	UF	-0.007	0.003	SF	-0.005	0.003	-0.002	0.004	0.879
InO and SoM	2m	SF	-0.005	0.003	UF	-0.007	0.003	0.002	0.004	0.879
InO and Vis	2m	MD	-0.016	0.005	SF	-0.006	0.005	-0.010	0.007	0.340
InO and Vis	2m	SF	-0.006	0.005	MD	-0.016	0.005	0.010	0.007	0.340
InO and Vis	2m	MD	-0.016	0.005	UF	-0.006	0.006	-0.010	0.008	0.424
InO and Vis	2m	UF	-0.006	0.006	MD	-0.016	0.005	0.010	0.008	0.424
InO and Vis	2m	SF	-0.006	0.005	UF	-0.006	0.006	-0.000	0.008	1.000
InO and Vis	2m	UF	-0.006	0.006	SF	-0.006	0.005	0.000	0.008	1.000
Lmb and Lmb	2m	MD	0.009	0.003	SF	0.007	0.003	0.002	0.005	0.938
Lmb and Lmb	2m	SF	0.007	0.003	MD	0.009	0.003	-0.002	0.005	0.938
Lmb and Lmb	2m	SF	0.007	0.003	UF	0.008	0.004	-0.001	0.005	0.976
Lmb and Lmb	2m	UF	0.008	0.004	SF	0.007	0.003	0.001	0.005	0.976
Lmb and Lmb	2m	MD	0.009	0.003	UF	0.008	0.004	0.000	0.005	0.996
Lmb and Lmb	2m	UF	0.008	0.004	MD	0.009	0.003	-0.000	0.005	0.996
Lmb and SoM	2m	MD	-0.010	0.002	UF	-0.006	0.002	-0.004	0.003	0.401
Lmb and SoM	2m	UF	-0.006	0.002	MD	-0.010	0.002	0.004	0.003	0.401
Lmb and SoM	2m	UF	-0.006	0.002	SF	-0.008	0.002	0.003	0.003	0.607
Lmb and SoM	2m	SF	-0.008	0.002	UF	-0.006	0.002	-0.003	0.003	0.607
Lmb and SoM	2m	MD	-0.010	0.002	SF	-0.008	0.002	-0.001	0.003	0.913
Lmb and SoM	2m	SF	-0.008	0.002	MD	-0.010	0.002	0.001	0.003	0.913
Lmb and Vis	2m	MD	-0.007	0.002	SF	-0.015	0.002	0.007	0.003	0.108
Lmb and Vis	2m	SF	-0.015	0.002	MD	-0.007	0.002	-0.007	0.003	0.108
Lmb and Vis	2m	SF	-0.015	0.002	UF	-0.009	0.003	-0.005	0.004	0.361
Lmb and Vis	2m	UF	-0.009	0.003	SF	-0.015	0.002	0.005	0.004	0.361
Lmb and Vis	2m	MD	-0.007	0.002	UF	-0.009	0.003	0.002	0.004	0.879
Lmb and Vis	2m	UF	-0.009	0.003	MD	-0.007	0.002	-0.002	0.004	0.879
SoM and SoM	2m	MD	0.007	0.004	SF	-0.003	0.004	0.010	0.005	0.192
SoM and SoM	2m	SF	-0.003	0.004	MD	0.007	0.004	-0.010	0.005	0.192
SoM and SoM	2m	MD	0.007	0.004	UF	0.001	0.005	0.005	0.006	0.664
SoM and SoM	2m	UF	0.001	0.005	MD	0.007	0.004	-0.005	0.006	0.664
SoM and SoM	2m	UF	0.001	0.005	SF	-0.003	0.004	0.004	0.006	0.753

SoM and SoM	2m	SF	-0.003	0.004	UF	0.001	0.005	-0.004	0.006	0.753
SoM and Vis	2m	UF	-0.010	0.005	SF	-0.017	0.004	0.007	0.007	0.528
SoM and Vis	2m	SF	-0.017	0.004	UF	-0.010	0.005	-0.007	0.007	0.528
SoM and Vis	2m	MD	-0.012	0.004	SF	-0.017	0.004	0.005	0.006	0.631
SoM and Vis	2m	SF	-0.017	0.004	MD	-0.012	0.004	-0.005	0.006	0.631
SoM and Vis	2m	MD	-0.012	0.004	UF	-0.010	0.005	-0.002	0.007	0.956
SoM and Vis	2m	UF	-0.010	0.005	MD	-0.012	0.004	0.002	0.007	0.956
Vis and Vis	2m	SF	-0.007	0.007	UF	0.012	0.008	-0.019	0.011	0.201
Vis and Vis	2m	UF	0.012	0.008	SF	-0.007	0.007	0.019	0.011	0.201
Vis and Vis	2m	MD	0.000	0.007	UF	0.012	0.008	-0.012	0.011	0.516
Vis and Vis	2m	UF	0.012	0.008	MD	0.000	0.007	0.012	0.011	0.516
Vis and Vis	2m	MD	0.000	0.007	SF	-0.007	0.007	0.007	0.009	0.728
Vis and Vis	2m	SF	-0.007	0.007	MD	0.000	0.007	-0.007	0.009	0.728
Aud and Aud	4m	SF	0.024	0.008	UF	0.049	0.010	-0.025	0.013	0.167
Aud and Aud	4m	UF	0.049	0.010	SF	0.024	0.008	0.025	0.013	0.167
Aud and Aud	4m	MD	0.043	0.008	SF	0.024	0.008	0.019	0.012	0.242
Aud and Aud	4m	SF	0.024	0.008	MD	0.043	0.008	-0.019	0.012	0.242
Aud and Aud	4m	MD	0.043	0.008	UF	0.049	0.010	-0.006	0.013	0.902
Aud and Aud	4m	UF	0.049	0.010	MD	0.043	0.008	0.006	0.013	0.902
Aud and Def	4m	UF	-0.007	0.003	SF	-0.003	0.003	-0.004	0.004	0.545
Aud and Def	4m	SF	-0.003	0.003	UF	-0.007	0.003	0.004	0.004	0.545
Aud and Def	4m	MD	-0.004	0.003	UF	-0.007	0.003	0.003	0.004	0.749
Aud and Def	4m	UF	-0.007	0.003	MD	-0.004	0.003	-0.003	0.004	0.749
Aud and Def	4m	MD	-0.004	0.003	SF	-0.003	0.003	-0.001	0.004	0.921
Aud and Def	4m	SF	-0.003	0.003	MD	-0.004	0.003	0.001	0.004	0.921
Aud and DoA	4m	MD	-0.005	0.003	SF	-0.011	0.003	0.006	0.005	0.410
Aud and DoA	4m	SF	-0.011	0.003	MD	-0.005	0.003	-0.006	0.005	0.410
Aud and DoA	4m	MD	-0.005	0.003	UF	-0.012	0.004	0.007	0.006	0.444
Aud and DoA	4m	UF	-0.012	0.004	MD	-0.005	0.003	-0.007	0.006	0.444
Aud and DoA	4m	SF	-0.011	0.003	UF	-0.012	0.004	0.001	0.006	0.995
Aud and DoA	4m	UF	-0.012	0.004	SF	-0.011	0.003	-0.001	0.006	0.995
Aud and InO	4m	MD	-0.005	0.004	UF	0.005	0.005	-0.009	0.007	0.342
Aud and InO	4m	UF	0.005	0.005	MD	-0.005	0.004	0.009	0.007	0.342
Aud and InO	4m	MD	-0.005	0.004	SF	0.001	0.004	-0.006	0.006	0.580
Aud and InO	4m	SF	0.001	0.004	MD	-0.005	0.004	0.006	0.006	0.580
Aud and InO	4m	SF	0.001	0.004	UF	0.005	0.005	-0.004	0.007	0.845
Aud and InO	4m	UF	0.005	0.005	SF	0.001	0.004	0.004	0.007	0.845
Aud and Lmb	4m	MD	-0.004	0.003	UF	-0.008	0.004	0.003	0.006	0.806
Aud and Lmb	4m	UF	-0.008	0.004	MD	-0.004	0.003	-0.003	0.006	0.806
Aud and Lmb	4m	SF	-0.005	0.003	UF	-0.008	0.004	0.002	0.006	0.900

Aud and Lmb	4m	UF	-0.008	0.004	SF	-0.005	0.003	-0.002	0.006	0.900
Aud and Lmb	4m	MD	-0.004	0.003	SF	-0.005	0.003	0.001	0.005	0.974
Aud and Lmb	4m	SF	-0.005	0.003	MD	-0.004	0.003	-0.001	0.005	0.974
Aud and SoM	4m	MD	0.000	0.003	UF	-0.004	0.004	0.005	0.005	0.626
Aud and SoM	4m	UF	-0.004	0.004	MD	0.000	0.003	-0.005	0.005	0.626
Aud and SoM	4m	MD	0.000	0.003	SF	-0.002	0.003	0.003	0.004	0.800
Aud and SoM	4m	SF	-0.002	0.003	MD	0.000	0.003	-0.003	0.004	0.800
Aud and SoM	4m	SF	-0.002	0.003	UF	-0.004	0.004	0.002	0.005	0.927
Aud and SoM	4m	UF	-0.004	0.004	SF	-0.002	0.003	-0.002	0.005	0.927
Aud and Vis	4m	MD	-0.017	0.008	SF	-0.009	0.008	-0.008	0.011	0.760
Aud and Vis	4m	SF	-0.009	0.008	MD	-0.017	0.008	0.008	0.011	0.760
Aud and Vis	4m	MD	-0.017	0.008	UF	-0.011	0.010	-0.006	0.013	0.902
Aud and Vis	4m	UF	-0.011	0.010	MD	-0.017	0.008	0.006	0.013	0.902
Aud and Vis	4m	SF	-0.009	0.008	UF	-0.011	0.010	0.002	0.013	0.980
Aud and Vis	4m	UF	-0.011	0.010	SF	-0.009	0.008	-0.002	0.013	0.980
Def and Def	4m	UF	0.003	0.002	SF	0.007	0.001	-0.003	0.002	0.347
Def and Def	4m	SF	0.007	0.001	UF	0.003	0.002	0.003	0.002	0.347
Def and Def	4m	MD	0.005	0.001	SF	0.007	0.001	-0.002	0.002	0.603
Def and Def	4m	SF	0.007	0.001	MD	0.005	0.001	0.002	0.002	0.603
Def and Def	4m	UF	0.003	0.002	MD	0.005	0.001	-0.001	0.002	0.834
Def and Def	4m	MD	0.005	0.001	UF	0.003	0.002	0.001	0.002	0.834
Def and DoA	4m	MD	-0.006	0.002	SF	-0.007	0.002	0.001	0.002	0.914
Def and DoA	4m	SF	-0.007	0.002	MD	-0.006	0.002	-0.001	0.002	0.914
Def and DoA	4m	MD	-0.006	0.002	UF	-0.006	0.002	0.001	0.003	0.970
Def and DoA	4m	UF	-0.006	0.002	MD	-0.006	0.002	-0.001	0.003	0.970
Def and DoA	4m	SF	-0.007	0.002	UF	-0.006	0.002	-0.000	0.003	0.992
Def and DoA	4m	UF	-0.006	0.002	SF	-0.007	0.002	0.000	0.003	0.992
Def and InO	4m	SF	-0.003	0.002	UF	-0.005	0.003	0.001	0.004	0.954
Def and InO	4m	UF	-0.005	0.003	SF	-0.003	0.002	-0.001	0.004	0.954
Def and InO	4m	MD	-0.004	0.002	UF	-0.005	0.003	0.001	0.004	0.982
Def and InO	4m	UF	-0.005	0.003	MD	-0.004	0.002	-0.001	0.004	0.982
Def and InO	4m	MD	-0.004	0.002	SF	-0.003	0.002	-0.000	0.003	0.991
Def and InO	4m	SF	-0.003	0.002	MD	-0.004	0.002	0.000	0.003	0.991
Def and Lmb	4m	UF	-0.003	0.001	MD	-0.001	0.001	-0.002	0.002	0.599
Def and Lmb	4m	MD	-0.001	0.001	UF	-0.003	0.001	0.002	0.002	0.599
Def and Lmb	4m	SF	-0.002	0.001	UF	-0.003	0.001	0.001	0.002	0.760
Def and Lmb	4m	UF	-0.003	0.001	SF	-0.002	0.001	-0.001	0.002	0.760
Def and Lmb	4m	MD	-0.001	0.001	SF	-0.002	0.001	0.000	0.002	0.950
Def and Lmb	4m	SF	-0.002	0.001	MD	-0.001	0.001	-0.000	0.002	0.950
Def and SoM	4m	MD	-0.007	0.001	SF	-0.005	0.001	-0.002	0.002	0.477

Def and SoM	4m	SF	-0.005	0.001	MD	-0.007	0.001	0.002	0.002	0.477
Def and SoM	4m	MD	-0.007	0.001	UF	-0.005	0.002	-0.002	0.002	0.645
Def and SoM	4m	UF	-0.005	0.002	MD	-0.007	0.001	0.002	0.002	0.645
Def and SoM	4m	SF	-0.005	0.001	UF	-0.005	0.002	0.000	0.002	0.990
Def and SoM	4m	UF	-0.005	0.002	SF	-0.005	0.001	-0.000	0.002	0.990
Def and Vis	4m	MD	-0.007	0.002	UF	-0.002	0.003	-0.005	0.003	0.390
Def and Vis	4m	UF	-0.002	0.003	MD	-0.007	0.002	0.005	0.003	0.390
Def and Vis	4m	MD	-0.007	0.002	SF	-0.004	0.002	-0.003	0.003	0.671
Def and Vis	4m	SF	-0.004	0.002	MD	-0.007	0.002	0.003	0.003	0.671
Def and Vis	4m	SF	-0.004	0.002	UF	-0.002	0.003	-0.002	0.003	0.828
Def and Vis	4m	UF	-0.002	0.003	SF	-0.004	0.002	0.002	0.003	0.828
DoA and DoA	4m	UF	0.005	0.006	MD	-0.003	0.005	0.008	0.008	0.554
DoA and DoA	4m	MD	-0.003	0.005	UF	0.005	0.006	-0.008	0.008	0.554
DoA and DoA	4m	SF	0.001	0.005	UF	0.005	0.006	-0.004	0.008	0.839
DoA and DoA	4m	UF	0.005	0.006	SF	0.001	0.005	0.004	0.008	0.839
DoA and DoA	4m	MD	-0.003	0.005	SF	0.001	0.005	-0.004	0.007	0.845
DoA and DoA	4m	SF	0.001	0.005	MD	-0.003	0.005	0.004	0.007	0.845
DoA and InO	4m	MD	-0.008	0.003	UF	-0.012	0.004	0.005	0.005	0.621
DoA and InO	4m	UF	-0.012	0.004	MD	-0.008	0.003	-0.005	0.005	0.621
DoA and InO	4m	MD	-0.008	0.003	SF	-0.011	0.003	0.003	0.004	0.750
DoA and InO	4m	SF	-0.011	0.003	MD	-0.008	0.003	-0.003	0.004	0.750
DoA and InO	4m	SF	-0.011	0.003	UF	-0.012	0.004	0.001	0.005	0.950
DoA and InO	4m	UF	-0.012	0.004	SF	-0.011	0.003	-0.001	0.005	0.950
DoA and Lmb	4m	MD	-0.005	0.002	SF	-0.009	0.002	0.004	0.003	0.340
DoA and Lmb	4m	SF	-0.009	0.002	MD	-0.005	0.002	-0.004	0.003	0.340
DoA and Lmb	4m	MD	-0.005	0.002	UF	-0.009	0.003	0.004	0.003	0.471
DoA and Lmb	4m	UF	-0.009	0.003	MD	-0.005	0.002	-0.004	0.003	0.471
DoA and Lmb	4m	SF	-0.009	0.002	UF	-0.009	0.003	-0.000	0.003	0.997
DoA and Lmb	4m	UF	-0.009	0.003	SF	-0.009	0.002	0.000	0.003	0.997
DoA and SoM	4m	MD	0.003	0.002	SF	-0.009	0.002	0.012	0.003	**0.005**
DoA and SoM	4m	SF	-0.009	0.002	MD	0.003	0.002	-0.012	0.003	**0.005**
DoA and SoM	4m	MD	0.003	0.002	UF	-0.006	0.003	0.009	0.004	0.053
DoA and SoM	4m	UF	-0.006	0.003	MD	0.003	0.002	-0.009	0.004	0.053
DoA and SoM	4m	SF	-0.009	0.002	UF	-0.006	0.003	-0.003	0.004	0.769
DoA and SoM	4m	UF	-0.006	0.003	SF	-0.009	0.002	0.003	0.004	0.769
DoA and Vis	4m	MD	-0.005	0.003	UF	-0.013	0.003	0.007	0.004	0.196
DoA and Vis	4m	UF	-0.013	0.003	MD	-0.005	0.003	-0.007	0.004	0.196
DoA and Vis	4m	MD	-0.005	0.003	SF	-0.011	0.003	0.005	0.004	0.297
DoA and Vis	4m	SF	-0.011	0.003	MD	-0.005	0.003	-0.005	0.004	0.297
DoA and Vis	4m	SF	-0.011	0.003	UF	-0.013	0.003	0.002	0.004	0.893

DoA and Vis	4m	UF	-0.013	0.003	SF	-0.011	0.003	-0.002	0.004	0.893
InO and InO	4m	MD	0.005	0.006	UF	0.016	0.008	-0.011	0.010	0.557
InO and InO	4m	UF	0.016	0.008	MD	0.005	0.006	0.011	0.010	0.557
InO and InO	4m	MD	0.005	0.006	SF	0.013	0.006	-0.007	0.009	0.710
InO and InO	4m	SF	0.013	0.006	MD	0.005	0.006	0.007	0.009	0.710
InO and InO	4m	UF	0.016	0.008	SF	0.013	0.006	0.004	0.010	0.935
InO and InO	4m	SF	0.013	0.006	UF	0.016	0.008	-0.004	0.010	0.935
InO and Lmb	4m	UF	-0.009	0.003	MD	-0.001	0.003	-0.008	0.004	0.153
InO and Lmb	4m	MD	-0.001	0.003	UF	-0.009	0.003	0.008	0.004	0.153
InO and Lmb	4m	MD	-0.001	0.003	SF	-0.005	0.003	0.004	0.004	0.532
InO and Lmb	4m	SF	-0.005	0.003	MD	-0.001	0.003	-0.004	0.004	0.532
InO and Lmb	4m	SF	-0.005	0.003	UF	-0.009	0.003	0.004	0.004	0.588
InO and Lmb	4m	UF	-0.009	0.003	SF	-0.005	0.003	-0.004	0.004	0.588
InO and SoM	4m	MD	-0.004	0.003	SF	-0.006	0.003	0.002	0.004	0.869
InO and SoM	4m	SF	-0.006	0.003	MD	-0.004	0.003	-0.002	0.004	0.869
InO and SoM	4m	SF	-0.006	0.003	UF	-0.005	0.004	-0.001	0.005	0.958
InO and SoM	4m	UF	-0.005	0.004	SF	-0.006	0.003	0.001	0.005	0.958
InO and SoM	4m	MD	-0.004	0.003	UF	-0.005	0.004	0.001	0.005	0.985
InO and SoM	4m	UF	-0.005	0.004	MD	-0.004	0.003	-0.001	0.005	0.985
InO and Vis	4m	MD	-0.001	0.003	SF	-0.012	0.003	0.011	0.005	0.106
InO and Vis	4m	SF	-0.012	0.003	MD	-0.001	0.003	-0.011	0.005	0.106
InO and Vis	4m	MD	-0.001	0.003	UF	-0.012	0.004	0.011	0.006	0.172
InO and Vis	4m	UF	-0.012	0.004	MD	-0.001	0.003	-0.011	0.006	0.172
InO and Vis	4m	SF	-0.012	0.003	UF	-0.012	0.004	-0.000	0.006	1.000
InO and Vis	4m	UF	-0.012	0.004	SF	-0.012	0.003	0.000	0.006	1.000
Lmb and Lmb	4m	MD	0.003	0.004	UF	0.009	0.005	-0.006	0.006	0.585
Lmb and Lmb	4m	UF	0.009	0.005	MD	0.003	0.004	0.006	0.006	0.585
Lmb and Lmb	4m	SF	0.005	0.004	UF	0.009	0.005	-0.003	0.006	0.849
Lmb and Lmb	4m	UF	0.009	0.005	SF	0.005	0.004	0.003	0.006	0.849
Lmb and Lmb	4m	MD	0.003	0.004	SF	0.005	0.004	-0.003	0.005	0.863
Lmb and Lmb	4m	SF	0.005	0.004	MD	0.003	0.004	0.003	0.005	0.863
Lmb and SoM	4m	MD	-0.010	0.002	UF	-0.006	0.002	-0.003	0.003	0.570
Lmb and SoM	4m	UF	-0.006	0.002	MD	-0.010	0.002	0.003	0.003	0.570
Lmb and SoM	4m	SF	-0.009	0.002	UF	-0.006	0.002	-0.003	0.003	0.588
Lmb and SoM	4m	UF	-0.006	0.002	SF	-0.009	0.002	0.003	0.003	0.588
Lmb and SoM	4m	MD	-0.010	0.002	SF	-0.009	0.002	-0.000	0.003	0.999
Lmb and SoM	4m	SF	-0.009	0.002	MD	-0.010	0.002	0.000	0.003	0.999
Lmb and Vis	4m	MD	-0.012	0.004	UF	-0.010	0.005	-0.002	0.007	0.971
Lmb and Vis	4m	UF	-0.010	0.005	MD	-0.012	0.004	0.002	0.007	0.971
Lmb and Vis	4m	SF	-0.012	0.004	UF	-0.010	0.005	-0.001	0.007	0.988

Lmb and Vis	4m	UF	-0.010	0.005	SF	-0.012	0.004	0.001	0.007	0.988
Lmb and Vis	4m	MD	-0.012	0.004	SF	-0.012	0.004	-0.001	0.006	0.995
Lmb and Vis	4m	SF	-0.012	0.004	MD	-0.012	0.004	0.001	0.006	0.995
SoM and SoM	4m	SF	-0.007	0.003	UF	0.003	0.004	-0.010	0.005	0.176
SoM and SoM	4m	UF	0.003	0.004	SF	-0.007	0.003	0.010	0.005	0.176
SoM and SoM	4m	MD	-0.001	0.003	SF	-0.007	0.003	0.006	0.005	0.437
SoM and SoM	4m	SF	-0.007	0.003	MD	-0.001	0.003	-0.006	0.005	0.437
SoM and SoM	4m	MD	-0.001	0.003	UF	0.003	0.004	-0.004	0.005	0.726
SoM and SoM	4m	UF	0.003	0.004	MD	-0.001	0.003	0.004	0.005	0.726
SoM and Vis	4m	MD	-0.015	0.004	UF	-0.009	0.004	-0.006	0.006	0.576
SoM and Vis	4m	UF	-0.009	0.004	MD	-0.015	0.004	0.006	0.006	0.576
SoM and Vis	4m	MD	-0.015	0.004	SF	-0.011	0.004	-0.003	0.005	0.807
SoM and Vis	4m	SF	-0.011	0.004	MD	-0.015	0.004	0.003	0.005	0.807
SoM and Vis	4m	SF	-0.011	0.004	UF	-0.009	0.004	-0.003	0.006	0.888
SoM and Vis	4m	UF	-0.009	0.004	SF	-0.011	0.004	0.003	0.006	0.888
Vis and Vis	4m	MD	-0.005	0.006	SF	0.004	0.006	-0.009	0.009	0.567
Vis and Vis	4m	SF	0.004	0.006	MD	-0.005	0.006	0.009	0.009	0.567
Vis and Vis	4m	SF	0.004	0.006	UF	-0.005	0.008	0.010	0.010	0.599
Vis and Vis	4m	UF	-0.005	0.008	SF	0.004	0.006	-0.010	0.010	0.599
Vis and Vis	4m	MD	-0.005	0.006	UF	-0.005	0.008	0.001	0.010	0.997
Vis and Vis	4m	UF	-0.005	0.008	MD	-0.005	0.006	-0.001	0.010	0.997
Aud and Aud	6m	SF	0.039	0.012	UF	0.048	0.016	-0.008	0.020	0.906
Aud and Aud	6m	UF	0.048	0.016	SF	0.039	0.012	0.008	0.020	0.906
Aud and Aud	6m	MD	0.041	0.012	UF	0.048	0.016	-0.007	0.020	0.941
Aud and Aud	6m	UF	0.048	0.016	MD	0.041	0.012	0.007	0.020	0.941
Aud and Aud	6m	MD	0.041	0.012	SF	0.039	0.012	0.002	0.018	0.994
Aud and Aud	6m	SF	0.039	0.012	MD	0.041	0.012	-0.002	0.018	0.994
Aud and Def	6m	MD	-0.002	0.002	SF	-0.006	0.002	0.004	0.003	0.332
Aud and Def	6m	SF	-0.006	0.002	MD	-0.002	0.002	-0.004	0.003	0.332
Aud and Def	6m	MD	-0.002	0.002	UF	-0.006	0.002	0.004	0.003	0.428
Aud and Def	6m	UF	-0.006	0.002	MD	-0.002	0.002	-0.004	0.003	0.428
Aud and Def	6m	SF	-0.006	0.002	UF	-0.006	0.002	-0.000	0.003	1.000
Aud and Def	6m	UF	-0.006	0.002	SF	-0.006	0.002	0.000	0.003	1.000
Aud and DoA	6m	MD	-0.015	0.003	UF	-0.002	0.004	-0.013	0.005	**0.038**
Aud and DoA	6m	UF	-0.002	0.004	MD	-0.015	0.003	0.013	0.005	**0.038**
Aud and DoA	6m	MD	-0.015	0.003	SF	-0.008	0.003	-0.007	0.004	0.266
Aud and DoA	6m	SF	-0.008	0.003	MD	-0.015	0.003	0.007	0.004	0.266
Aud and DoA	6m	SF	-0.008	0.003	UF	-0.002	0.004	-0.006	0.005	0.429
Aud and DoA	6m	UF	-0.002	0.004	SF	-0.008	0.003	0.006	0.005	0.429
Aud and InO	6m	MD	0.004	0.005	SF	-0.002	0.005	0.006	0.006	0.646

Aud and InO	6m	SF	-0.002	0.005	MD	0.004	0.005	-0.006	0.006	0.646
Aud and InO	6m	SF	-0.002	0.005	UF	0.004	0.006	-0.006	0.007	0.711
Aud and InO	6m	UF	0.004	0.006	SF	-0.002	0.005	0.006	0.007	0.711
Aud and InO	6m	MD	0.004	0.005	UF	0.004	0.006	-0.000	0.007	1.000
Aud and InO	6m	UF	0.004	0.006	MD	0.004	0.005	0.000	0.007	1.000
Aud and Lmb	6m	MD	0.001	0.003	UF	-0.011	0.004	0.012	0.005	0.072
Aud and Lmb	6m	UF	-0.011	0.004	MD	0.001	0.003	-0.012	0.005	0.072
Aud and Lmb	6m	SF	-0.004	0.003	UF	-0.011	0.004	0.007	0.005	0.364
Aud and Lmb	6m	UF	-0.011	0.004	SF	-0.004	0.003	-0.007	0.005	0.364
Aud and Lmb	6m	MD	0.001	0.003	SF	-0.004	0.003	0.005	0.005	0.524
Aud and Lmb	6m	SF	-0.004	0.003	MD	0.001	0.003	-0.005	0.005	0.524
Aud and SoM	6m	MD	-0.003	0.004	UF	0.004	0.004	-0.007	0.006	0.450
Aud and SoM	6m	UF	0.004	0.004	MD	-0.003	0.004	0.007	0.006	0.450
Aud and SoM	6m	UF	0.004	0.004	SF	-0.000	0.004	0.004	0.006	0.718
Aud and SoM	6m	SF	-0.000	0.004	UF	0.004	0.004	-0.004	0.006	0.718
Aud and SoM	6m	MD	-0.003	0.004	SF	-0.000	0.004	-0.003	0.005	0.866
Aud and SoM	6m	SF	-0.000	0.004	MD	-0.003	0.004	0.003	0.005	0.866
Aud and Vis	6m	MD	-0.007	0.004	UF	-0.000	0.005	-0.007	0.006	0.475
Aud and Vis	6m	UF	-0.000	0.005	MD	-0.007	0.004	0.007	0.006	0.475
Aud and Vis	6m	MD	-0.007	0.004	SF	-0.004	0.004	-0.003	0.005	0.787
Aud and Vis	6m	SF	-0.004	0.004	MD	-0.007	0.004	0.003	0.005	0.787
Aud and Vis	6m	SF	-0.004	0.004	UF	-0.000	0.005	-0.004	0.006	0.819
Aud and Vis	6m	UF	-0.000	0.005	SF	-0.004	0.004	0.004	0.006	0.819
Def and Def	6m	MD	0.006	0.001	SF	0.004	0.001	0.002	0.002	0.543
Def and Def	6m	SF	0.004	0.001	MD	0.006	0.001	-0.002	0.002	0.543
Def and Def	6m	MD	0.006	0.001	UF	0.004	0.002	0.003	0.002	0.556
Def and Def	6m	UF	0.004	0.002	MD	0.006	0.001	-0.003	0.002	0.556
Def and Def	6m	SF	0.004	0.001	UF	0.004	0.002	0.000	0.002	0.993
Def and Def	6m	UF	0.004	0.002	SF	0.004	0.001	-0.000	0.002	0.993
Def and DoA	6m	MD	-0.006	0.001	SF	-0.004	0.001	-0.002	0.002	0.583
Def and DoA	6m	SF	-0.004	0.001	MD	-0.006	0.001	0.002	0.002	0.583
Def and DoA	6m	UF	-0.005	0.002	MD	-0.006	0.001	0.001	0.002	0.835
Def and DoA	6m	MD	-0.006	0.001	UF	-0.005	0.002	-0.001	0.002	0.835
Def and DoA	6m	SF	-0.004	0.001	UF	-0.005	0.002	0.001	0.002	0.949
Def and DoA	6m	UF	-0.005	0.002	SF	-0.004	0.001	-0.001	0.002	0.949
Def and InO	6m	MD	-0.001	0.002	SF	-0.006	0.002	0.004	0.003	0.259
Def and InO	6m	SF	-0.006	0.002	MD	-0.001	0.002	-0.004	0.003	0.259
Def and InO	6m	MD	-0.001	0.002	UF	-0.004	0.002	0.003	0.003	0.587
Def and InO	6m	UF	-0.004	0.002	MD	-0.001	0.002	-0.003	0.003	0.587
Def and InO	6m	SF	-0.006	0.002	UF	-0.004	0.002	-0.001	0.003	0.903

Def and InO	6m	UF	-0.004	0.002	SF	-0.006	0.002	0.001	0.003	0.903
Def and Lmb	6m	MD	0.001	0.001	SF	-0.001	0.001	0.002	0.002	0.559
Def and Lmb	6m	SF	-0.001	0.001	MD	0.001	0.001	-0.002	0.002	0.559
Def and Lmb	6m	UF	-0.001	0.001	MD	0.001	0.001	-0.002	0.002	0.660
Def and Lmb	6m	MD	0.001	0.001	UF	-0.001	0.001	0.002	0.002	0.660
Def and Lmb	6m	SF	-0.001	0.001	UF	-0.001	0.001	-0.000	0.002	0.999
Def and Lmb	6m	UF	-0.001	0.001	SF	-0.001	0.001	0.000	0.002	0.999
Def and SoM	6m	MD	-0.007	0.001	UF	-0.009	0.002	0.003	0.002	0.508
Def and SoM	6m	UF	-0.009	0.002	MD	-0.007	0.001	-0.003	0.002	0.508
Def and SoM	6m	MD	-0.007	0.001	SF	-0.008	0.001	0.001	0.002	0.791
Def and SoM	6m	SF	-0.008	0.001	MD	-0.007	0.001	-0.001	0.002	0.791
Def and SoM	6m	SF	-0.008	0.001	UF	-0.009	0.002	0.001	0.002	0.846
Def and SoM	6m	UF	-0.009	0.002	SF	-0.008	0.001	-0.001	0.002	0.846
Def and Vis	6m	SF	-0.006	0.002	UF	-0.010	0.003	0.004	0.003	0.549
Def and Vis	6m	UF	-0.010	0.003	SF	-0.006	0.002	-0.004	0.003	0.549
Def and Vis	6m	MD	-0.008	0.002	SF	-0.006	0.002	-0.002	0.003	0.826
Def and Vis	6m	SF	-0.006	0.002	MD	-0.008	0.002	0.002	0.003	0.826
Def and Vis	6m	UF	-0.010	0.003	MD	-0.008	0.002	-0.002	0.003	0.852
Def and Vis	6m	MD	-0.008	0.002	UF	-0.010	0.003	0.002	0.003	0.852
DoA and DoA	6m	UF	0.003	0.004	SF	0.001	0.003	0.002	0.005	0.946
DoA and DoA	6m	SF	0.001	0.003	UF	0.003	0.004	-0.002	0.005	0.946
DoA and DoA	6m	MD	0.003	0.003	SF	0.001	0.003	0.001	0.005	0.969
DoA and DoA	6m	SF	0.001	0.003	MD	0.003	0.003	-0.001	0.005	0.969
DoA and DoA	6m	MD	0.003	0.003	UF	0.003	0.004	-0.001	0.005	0.994
DoA and DoA	6m	UF	0.003	0.004	MD	0.003	0.003	0.001	0.005	0.994
DoA and InO	6m	UF	-0.003	0.003	SF	-0.009	0.002	0.006	0.004	0.244
DoA and InO	6m	SF	-0.009	0.002	UF	-0.003	0.003	-0.006	0.004	0.244
DoA and InO	6m	MD	-0.006	0.002	SF	-0.009	0.002	0.003	0.003	0.635
DoA and InO	6m	SF	-0.009	0.002	MD	-0.006	0.002	-0.003	0.003	0.635
DoA and InO	6m	UF	-0.003	0.003	MD	-0.006	0.002	0.003	0.004	0.671
DoA and InO	6m	MD	-0.006	0.002	UF	-0.003	0.003	-0.003	0.004	0.671
DoA and Lmb	6m	SF	-0.007	0.001	UF	-0.007	0.002	-0.001	0.002	0.920
DoA and Lmb	6m	UF	-0.007	0.002	SF	-0.007	0.001	0.001	0.002	0.920
DoA and Lmb	6m	MD	-0.007	0.001	UF	-0.007	0.002	-0.001	0.002	0.965
DoA and Lmb	6m	UF	-0.007	0.002	MD	-0.007	0.001	0.001	0.002	0.965
DoA and Lmb	6m	MD	-0.007	0.001	SF	-0.007	0.001	0.000	0.002	0.987
DoA and Lmb	6m	SF	-0.007	0.001	MD	-0.007	0.001	-0.000	0.002	0.987
DoA and SoM	6m	MD	0.000	0.003	SF	-0.004	0.003	0.005	0.004	0.433
DoA and SoM	6m	SF	-0.004	0.003	MD	0.000	0.003	-0.005	0.004	0.433
DoA and SoM	6m	MD	0.000	0.003	UF	-0.002	0.003	0.003	0.004	0.813

DoA and SoM	6m	UF	-0.002	0.003	MD	0.000	0.003	-0.003	0.004	0.813
DoA and SoM	6m	UF	-0.002	0.003	SF	-0.004	0.003	0.002	0.004	0.876
DoA and SoM	6m	SF	-0.004	0.003	UF	-0.002	0.003	-0.002	0.004	0.876
DoA and Vis	6m	MD	-0.008	0.003	UF	-0.006	0.003	-0.003	0.004	0.768
DoA and Vis	6m	UF	-0.006	0.003	MD	-0.008	0.003	0.003	0.004	0.768
DoA and Vis	6m	MD	-0.008	0.003	SF	-0.007	0.003	-0.002	0.004	0.877
DoA and Vis	6m	SF	-0.007	0.003	MD	-0.008	0.003	0.002	0.004	0.877
DoA and Vis	6m	SF	-0.007	0.003	UF	-0.006	0.003	-0.001	0.004	0.961
DoA and Vis	6m	UF	-0.006	0.003	SF	-0.007	0.003	0.001	0.004	0.961
InO and InO	6m	MD	0.047	0.008	SF	0.025	0.008	0.021	0.011	0.154
InO and InO	6m	SF	0.025	0.008	MD	0.047	0.008	-0.021	0.011	0.154
InO and InO	6m	MD	0.047	0.008	UF	0.026	0.010	0.021	0.013	0.232
InO and InO	6m	UF	0.026	0.010	MD	0.047	0.008	-0.021	0.013	0.232
InO and InO	6m	SF	0.025	0.008	UF	0.026	0.010	-0.000	0.013	1.000
InO and InO	6m	UF	0.026	0.010	SF	0.025	0.008	0.000	0.013	1.000
InO and Lmb	6m	SF	-0.004	0.002	UF	-0.001	0.002	-0.002	0.003	0.750
InO and Lmb	6m	UF	-0.001	0.002	SF	-0.004	0.002	0.002	0.003	0.750
InO and Lmb	6m	MD	-0.002	0.002	SF	-0.004	0.002	0.002	0.003	0.829
InO and Lmb	6m	SF	-0.004	0.002	MD	-0.002	0.002	-0.002	0.003	0.829
InO and Lmb	6m	MD	-0.002	0.002	UF	-0.001	0.002	-0.001	0.003	0.975
InO and Lmb	6m	UF	-0.001	0.002	MD	-0.002	0.002	0.001	0.003	0.975
InO and SoM	6m	MD	-0.001	0.002	UF	-0.006	0.002	0.005	0.003	0.303
InO and SoM	6m	UF	-0.006	0.002	MD	-0.001	0.002	-0.005	0.003	0.303
InO and SoM	6m	MD	-0.001	0.002	SF	-0.004	0.002	0.003	0.003	0.470
InO and SoM	6m	SF	-0.004	0.002	MD	-0.001	0.002	-0.003	0.003	0.470
InO and SoM	6m	SF	-0.004	0.002	UF	-0.006	0.002	0.001	0.003	0.883
InO and SoM	6m	UF	-0.006	0.002	SF	-0.004	0.002	-0.001	0.003	0.883
InO and Vis	6m	SF	-0.012	0.005	UF	-0.011	0.006	-0.000	0.008	0.998
InO and Vis	6m	UF	-0.011	0.006	SF	-0.012	0.005	0.000	0.008	0.998
InO and Vis	6m	MD	-0.011	0.005	SF	-0.012	0.005	0.000	0.007	0.999
InO and Vis	6m	SF	-0.012	0.005	MD	-0.011	0.005	-0.000	0.007	0.999
InO and Vis	6m	MD	-0.011	0.005	UF	-0.011	0.006	-0.000	0.008	1.000
InO and Vis	6m	UF	-0.011	0.006	MD	-0.011	0.005	0.000	0.008	1.000
Lmb and Lmb	6m	MD	0.012	0.004	SF	0.004	0.004	0.008	0.005	0.314
Lmb and Lmb	6m	SF	0.004	0.004	MD	0.012	0.004	-0.008	0.005	0.314
Lmb and Lmb	6m	MD	0.012	0.004	UF	0.007	0.005	0.005	0.006	0.684
Lmb and Lmb	6m	UF	0.007	0.005	MD	0.012	0.004	-0.005	0.006	0.684
Lmb and Lmb	6m	SF	0.004	0.004	UF	0.007	0.005	-0.003	0.006	0.883
Lmb and Lmb	6m	UF	0.007	0.005	SF	0.004	0.004	0.003	0.006	0.883
Lmb and SoM	6m	MD	-0.006	0.003	UF	-0.011	0.003	0.005	0.004	0.469

Lmb and SoM	6m	UF	-0.011	0.003	MD	-0.006	0.003	-0.005	0.004	0.469
Lmb and SoM	6m	MD	-0.006	0.003	SF	-0.009	0.003	0.004	0.004	0.623
Lmb and SoM	6m	SF	-0.009	0.003	MD	-0.006	0.003	-0.004	0.004	0.623
Lmb and SoM	6m	SF	-0.009	0.003	UF	-0.011	0.003	0.002	0.004	0.926
Lmb and SoM	6m	UF	-0.011	0.003	SF	-0.009	0.003	-0.002	0.004	0.926
Lmb and Vis	6m	MD	-0.007	0.003	UF	-0.016	0.003	0.009	0.004	0.124
Lmb and Vis	6m	UF	-0.016	0.003	MD	-0.007	0.003	-0.009	0.004	0.124
Lmb and Vis	6m	SF	-0.010	0.003	UF	-0.016	0.003	0.005	0.004	0.435
Lmb and Vis	6m	UF	-0.016	0.003	SF	-0.010	0.003	-0.005	0.004	0.435
Lmb and Vis	6m	MD	-0.007	0.003	SF	-0.010	0.003	0.003	0.004	0.632
Lmb and Vis	6m	SF	-0.010	0.003	MD	-0.007	0.003	-0.003	0.004	0.632
SoM and SoM	6m	MD	0.008	0.004	UF	-0.014	0.005	0.021	0.006	**0.005**
SoM and SoM	6m	UF	-0.014	0.005	MD	0.008	0.004	-0.021	0.006	**0.005**
SoM and SoM	6m	SF	0.005	0.004	UF	-0.014	0.005	0.019	0.006	**0.011**
SoM and SoM	6m	UF	-0.014	0.005	SF	0.005	0.004	-0.019	0.006	**0.011**
SoM and SoM	6m	MD	0.008	0.004	SF	0.005	0.004	0.002	0.005	0.882
SoM and SoM	6m	SF	0.005	0.004	MD	0.008	0.004	-0.002	0.005	0.882
SoM and Vis	6m	SF	-0.011	0.002	UF	-0.025	0.003	0.014	0.004	**0.005**
SoM and Vis	6m	UF	-0.025	0.003	SF	-0.011	0.002	-0.014	0.004	**0.005**
SoM and Vis	6m	MD	-0.014	0.002	UF	-0.025	0.003	0.011	0.004	**0.021**
SoM and Vis	6m	UF	-0.025	0.003	MD	-0.014	0.002	-0.011	0.004	**0.021**
SoM and Vis	6m	MD	-0.014	0.002	SF	-0.011	0.002	-0.003	0.003	0.728
SoM and Vis	6m	SF	-0.011	0.002	MD	-0.014	0.002	0.003	0.003	0.728
Vis and Vis	6m	MD	0.010	0.003	SF	0.005	0.003	0.006	0.005	0.484
Vis and Vis	6m	SF	0.005	0.003	MD	0.010	0.003	-0.006	0.005	0.484
Vis and Vis	6m	MD	0.010	0.003	UF	0.006	0.004	0.004	0.005	0.700
Vis and Vis	6m	UF	0.006	0.004	MD	0.010	0.003	-0.004	0.005	0.700
Vis and Vis	6m	SF	0.005	0.003	UF	0.006	0.004	-0.001	0.005	0.975
Vis and Vis	6m	UF	0.006	0.004	SF	0.005	0.003	0.001	0.005	0.975

Figure S1

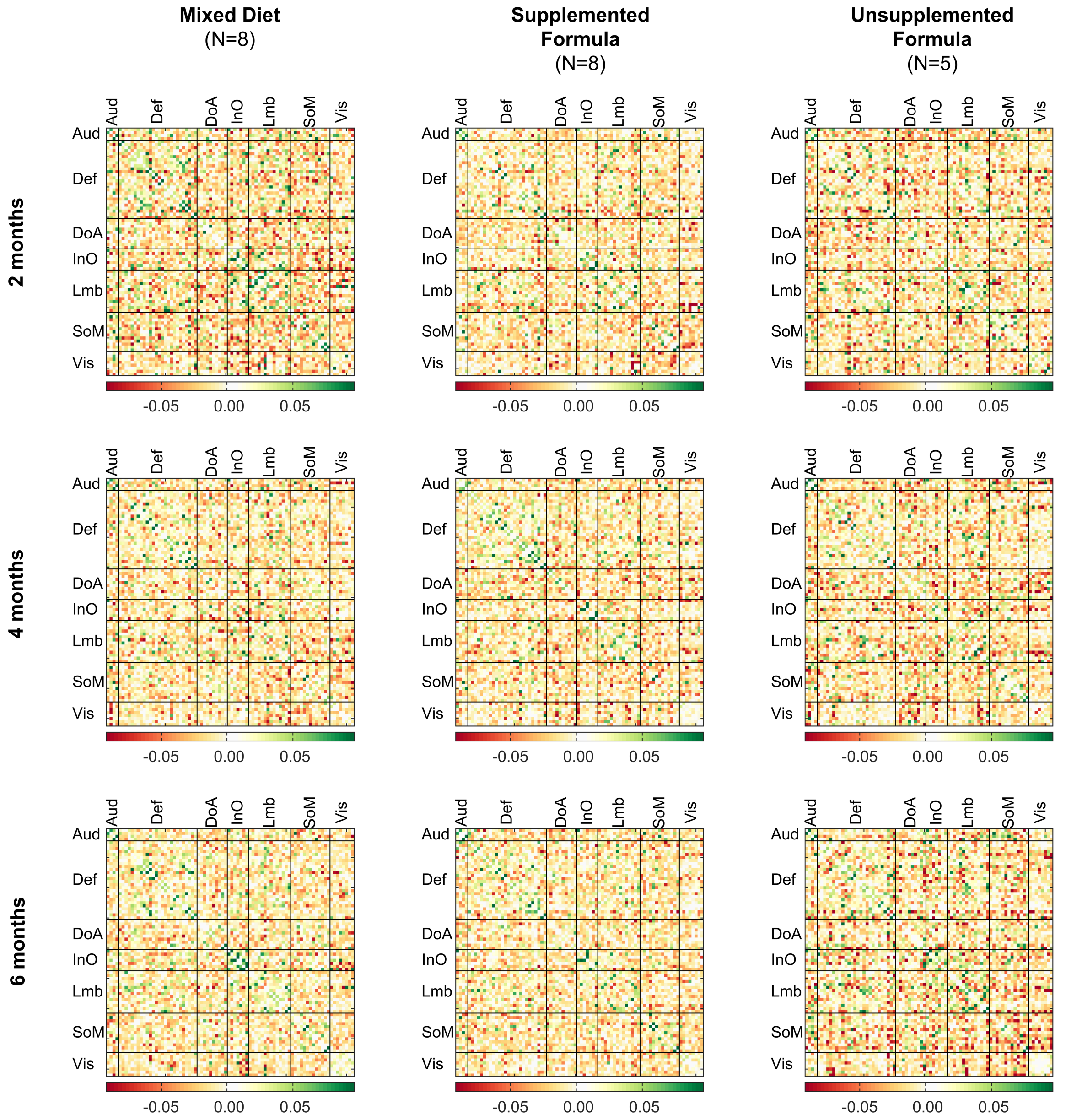
Mixed Diet
(N=8)
Supplemented Formula
(N=8)
Unsupplemented Formula
(N=5)
2 months
4 months
6 months
Aud
Def
DoA
InO
Lmb
SoM
Vis
-0.05
0.00
0.05

Figure S2

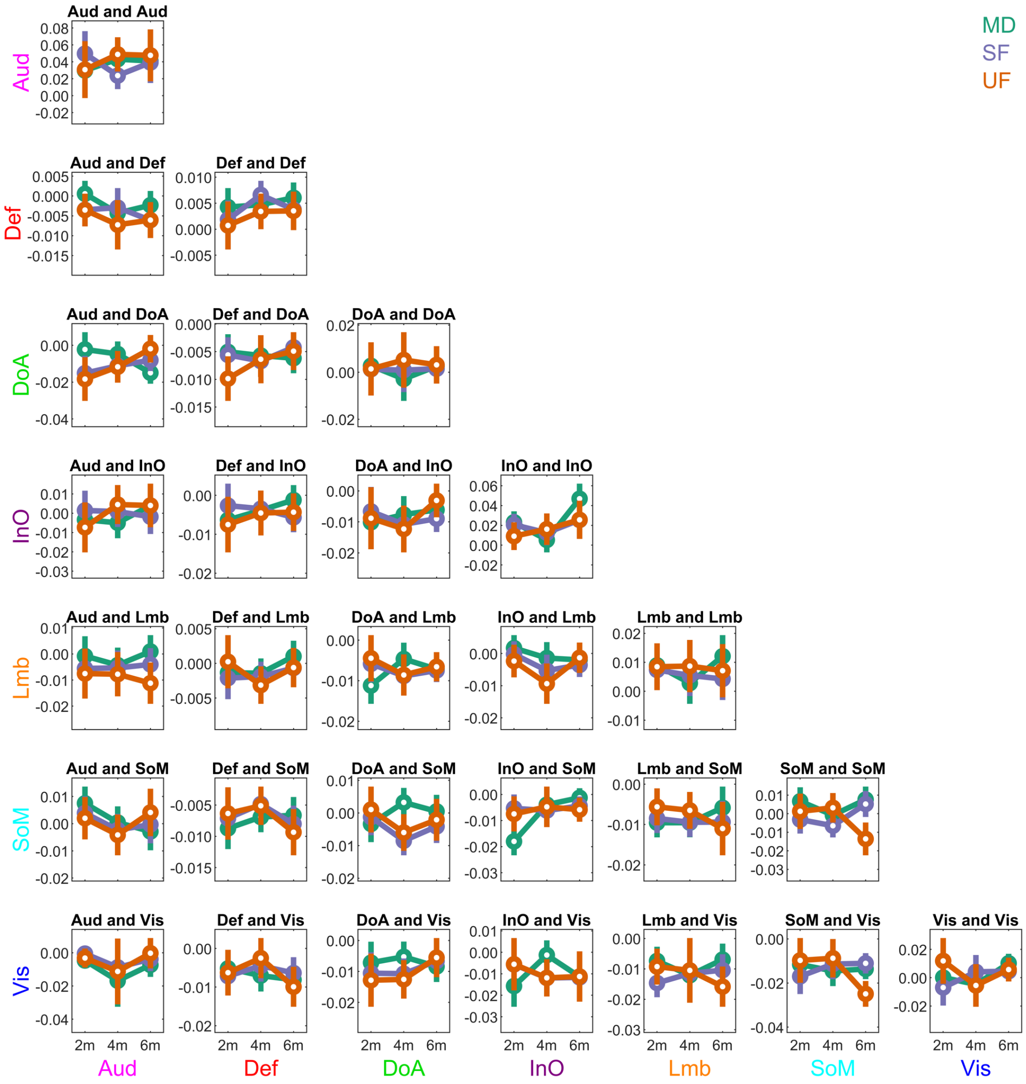
MD
SF
UF
Aud and Aud
Aud and Def
Def and Def
Aud and DoA
Def and DoA
DoA and DoA
Aud and InO
Def and InO
DoA and InO
InO and InO
Aud and Lmb
Def and Lmb
DoA and Lmb
InO and Lmb
Lmb and Lmb
Aud and SoM
Def and SoM
DoA and SoM
InO and SoM
Lmb and SoM
SoM and SoM
Aud and Vis
Def and Vis
DoA and Vis
InO and Vis
Lmb and Vis
SoM and Vis
Vis and Vis
Aud
Def
DoA
InO
Lmb
SoM
Vis
2m 4m 6m
